# Coenzyme Q10 mitigates macrophage mediated inflammation in heart following myocardial infarction via the NLRP3/IL1β pathway

**DOI:** 10.1186/s12872-024-03729-x

**Published:** 2024-01-28

**Authors:** Wenxu Pan, Guiquan Zhou, Meiling Hu, Gaoshan Li, Mingle Zhang, Hao Yang, Kunyan Li, Jingwei Li, Ting Liu, Ying Wang, Jun Jin

**Affiliations:** Department of Cardiology, The Second Affiliated Hospital of Army Medical University, Chongqing, China

**Keywords:** Coenzyme Q10, Macrophage, Inflammation, Myocardial infarction, Interleukin-1 beta

## Abstract

**Background:**

The protective effect of Coenzyme Q10 (CoQ10) on the cardiovascular system has been reported, however, whether it can promote early recovery of cardiac function and alleviate cardiac remodeling after myocardial infarction (MI) remains to be elucidated. Whether CoQ10 may regulate the macrophage-mediated pro-inflammatory response after MI and its potential mechanism are worth further exploration.

**Methods:**

To determine the baseline plasma levels of CoQ10 by LC-MS/MS, healthy controls and MI patients (*n* = 11 each) with age- and gender-matched were randomly enrolled. Additional MI patients were consecutively enrolled and randomized into the blank control (*n* = 59) or CoQ10 group (*n* = 61). Follow-ups were performed at 1- and 3-month to assess cardiac function after percutaneous coronary intervention (PCI). In the animal study, mice were orally administered CoQ10/vehicle daily and were subjected to left anterior descending coronary artery (LAD) ligation or sham operation. Echocardiography and serum BNP measured by ELISA were analyzed to evaluate cardiac function. Masson staining and WGA staining were performed to analyze the myocardial fibrosis and cardiomyocyte hypertrophy, respectively. Immunofluorescence staining was performed to assess the infiltration of IL1β/ROS-positive macrophages into the ischemic myocardium. Flow cytometry was employed to analyze the recruitment of myeloid immune cells to the ischemic myocardium post-MI. The expression of inflammatory indicators was assessed through RNA-seq, qPCR, and western blotting (WB).

**Results:**

Compared to controls, MI patients showed a plasma deficiency of CoQ10 (0.76 ± 0.31 vs. 0.46 ± 0.10 µg/ml). CoQ10 supplementation significantly promoted the recovery of cardiac function in MI patients at 1 and 3 months after PCI. In mice study, compared to vehicle-treated MI mice, CoQ10-treated MI mice showed a favorable trend in survival rate (42.85% vs. 61.90%), as well as significantly alleviated cardiac dysfunction, myocardial fibrosis, and cardiac hypertrophy. Notably, CoQ10 administration significantly suppressed the recruitment of pro-inflammatory CCR2^+^ macrophages into infarct myocardium and their mediated inflammatory response, partially by attenuating the activation of the NLR family pyrin domain containing 3 (NLRP3)/Interleukin-1 beta (IL1β) signaling pathway.

**Conclusions:**

These findings suggest that CoQ10 can significantly promote early recovery of cardiac function after MI. CoQ10 may function by inhibiting the recruitment of CCR2^+^ macrophages and suppressing the activation of the NLRP3/IL1β pathway in macrophages.

**Trial registration:**

Date of registration 09/04/2021 (number: ChiCTR2100045256).

**Supplementary Information:**

The online version contains supplementary material available at 10.1186/s12872-024-03729-x.

## Introduction

Myocardial infarction (MI) is the most severe consequence of coronary artery disease and a leading cause of morbidity and mortality worldwide [1, 2]. Despite strategies of revascularization and medications that have substantially reduced the mortality rate of MI, many patients still suffer from adverse cardiac remodeling and even progress to fatal heart failure (HF) [3–5]. In recent years, it has been increasingly appreciated that immune cells, especially macrophage-mediated inflammatory responses, play a critical role in the progression and prognosis of ischemic heart disease [6, 7]. The ischemic microenvironment induces the massive production of reactive oxygen species (ROS), which triggers the activation of the NOD-like receptor family pyrin domain-containing 3 (NLRP3) inflammasome and the release of the proinflammatory cytokine interleukin-1 β (IL1β) [8, 9]. Meanwhile, excessive production of IL1β promotes oxidative stress and ROS generation, forming a positive feedback loop that exacerbates cardiac inflammation [10]. Notably, all clinical trials that obtained significant cardiovascular benefits (CANTOS, COLCOT, and LoDoCo2) so far in terms of anti-inflammatory treatment for coronary heart disease target the NLRP3/IL1β inflammatory axis [11–13]. This suggests that NLRP3/IL1β may be important for effective anti-inflammatory therapy as well as further improving the prognosis of ischemic heart disease [14, 15]. However, the expensive price of canakinumab or the potential side effects of long-term use of colchicine (which include diarrhea and nausea) [11, 16] may affect the actual clinical application of these drugs.

Coenzyme Q10 (CoQ10) is a ubiquinone naturally present in animals and humans with multiple biological effects, such as promoting ATP generation and scavenging free oxygen radicals [17–20]. Moreover, as CoQ10 is synthesized through the mevalonate pathway, it also yields cholesterol as a by-product. Hence, aside from aging, drugs that inhibit the mevalonate pathway, like statins, have been reported to reduce the levels of CoQ10 in blood and tissues [21, 22]. According to clinical trials and meta-analyses focusing on CoQ10, supplementation with CoQ10 was found to effectively reduce adverse cardiovascular events in HF patientsand significantly lower the risk of all-cause mortality, especially due to ischemic origin [23–26]. However, although inflammation is the key mediator of MI prognosis, it remains unclear whether CoQ10 can improve the prognosis of MI by regulating the cardiac inflammatory response.

Therefore, in this study, we conducted a clinical and animal study to investigate the impact of CoQ10 on MI prognosis, with a particular focus on inflammation.

## Methods

### Clinical study

**Fig. 1:**
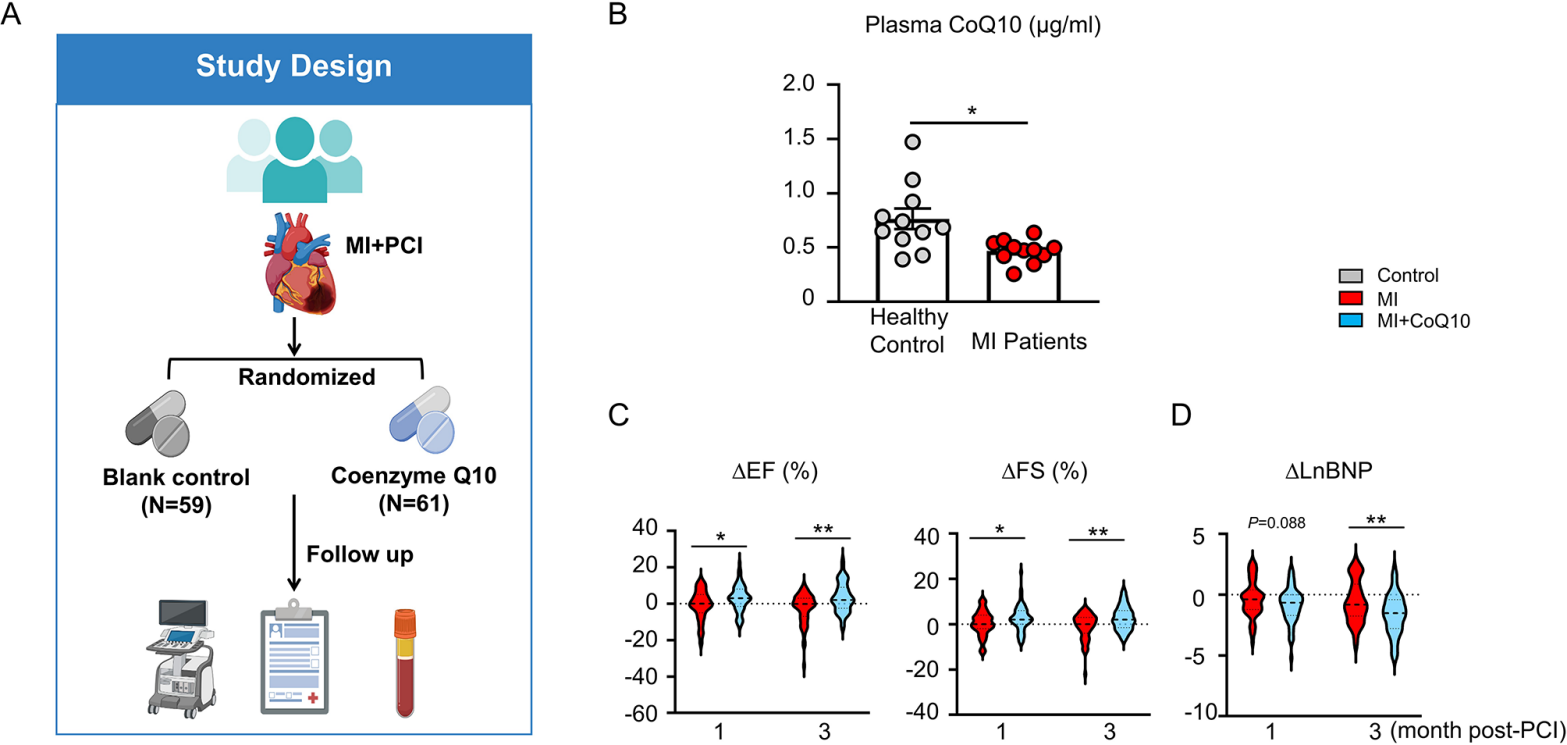
Plasma levels of CoQ10 are lower in MI patients (administrated with stains) compared to those in healthy controls, and supplementation with CoQ10 accelerates the recovery of cardiac function in MI patients after PCI therapy. **(A)** Illustration of the study design of the clinical experiment. **(B)** The baseline concentration of plasma CoQ10 of healthy controls and MI patients were analyzed by liquid chromatographic-tandem mass spectra-metric (LC-MS/MS) (Statistical analysis was performed using a two-tailed unpaired Student’s t-test). **(C)** Sequential echocardiographic analyses showed that MI patients supplemented with CoQ10 display a better recovery of cardiac function at 1-month and 3-month post-PCI compared with MI patients without CoQ10 treatment (Statistical analysis was performed using a Mann-Whitney U test). **(D)** The LnBNP of patients in the MI group and MI + CoQ10 group at 3-month post-PCI demonstrated a greater reduction in MI patients supplemented with CoQ10 compared with MI patients without CoQ10 treatment (Statistical analysis was performed using a Mann-Whitney U test). Grey indicates the control group (healthy volunteers); Red indicates the blank control group (MI patients); Blue indicates the CoQ10 group (MI + CoQ10). MI: myocardial infarction; PCI: percutaneous coronary intervention; CoQ10: Coenzyme Q10; EF: ejection fraction; FS: fractional shortening; BNP: brain natriuretic peptide. *p < 0.05, **p < 0.01

To measure the baseline plasma level of CoQ10, 11 MI patients (administrated with statins and dual antiplatelet therapy) and 11 healthy controls matched by age and gender were randomly enrolled (Supplemental Fig. 1A). Subjects who received warfarin, antioxidant vitamin, or CoQ10 treatment within the past three months were excluded from our survey. In addition, to investigate the impact of CoQ10 supplementation on the recovery of cardiac function in MI patients, we conducted an interim analysis of our ongoing clinical trial (a prospective, randomized, open-label, single-center clinical trial). 147 MI patients successfully treated with percutaneous coronary intervention (PCI) therapy were consecutively enrolled in this study from an ongoing clinical cohort (Supplemental Fig. 1B). All patients received standard acute coronary syndrome (ACS) medications and were then randomized into the blank control group (*n* = 71) or CoQ10 group (30 mg/d, Eisai, H10930021, *n* = 76) after PCI. Clinical and general parameters were recorded. Cardiac function parameters, including EF (ejection fraction, %) and FS (fractional shortening, %), as well as the HF parameter plasma BNP (brain natriuretic peptide) levels, were monitored at 1-month and 3-month follow-ups. Exclusion of the patients without echocardiography data at 1-month and 3-month follow-ups. Finally, 120 MI patients were included in the final analysis [blank control group (*n* = 59) and CoQ10 group (*n* = 61)] (Fig. 1A, Supplemental Fig. 1B). This study protocol was approved by the Ethics Committee of Xinqiao Hospital, Army Medical University (2021-041-01), and the trial was first registered in the Chinese Clinical Trial Registry (ChiCTR2100045256) on 09/04/2021. The first patient was enrolled on April 19, 2021. All participants were informed of the research contents and signed informed consent.

### Mice

8-10-week-old C57BL/6 mice were used in this study and were purchased from Beijing Vital River Laboratory Animal Technology Co. Ltd. and Beijing HFK Bioscience Co. Ltd. All mice were maintained on a 12-hour light-dark schedule in a specific pathogen-free animal facility. Food and water were given ad libitum. Male mice were used for the in vivo and ex vivo experiments. The number of mice included in each experiment is indicated in the Supplementary Materials. The Laboratory Animal Welfare and Ethics Committee of the Army Medical University approved all the animal procedures. All methods are reported in accordance with ARRIVE guidelines (https://arriveguidelines.org) for the reporting of animal experiments.

### CoQ10 supplementation

In the clinical study, MI patients were administered CoQ10 (Eisai, H10930021) orally at a dose of 30 mg/d. In the mice study, we determined the optimal method of CoQ10 (MCE, HY-N0111) administration as 600 mg/kg/d (dissolved in corn oil) by oral gavage (Supplemental Fig. 2). Mice were administered with freshly made CoQ10 or vehicle every day from 3 days prior to the surgeries until the end of the experiments. Plasma concentrations of CoQ10 were detected using the LC-MS/MS method as previously described [27].

### Experimental LAD ligation

Surgery was performed as we previously described [28, 29]. Briefly, the mice were anesthetized by inhalation of isoflurane (5% at the induction stage, 1% at the maintenance stage). After tracheal intubation, the chest was opened by a small incision from the 4th intercostal space. LAD was ligated tightly with a tapered 8 − 0 vicryl suture (J401G, Ethicon) under microscopy. Mice in the sham group obtained the same operation but no LAD ligation. Myocardial ischemia was confirmed by ST-T segment elevation in the electrocardiogram and the color changes of cardiac tissues in the segment of the left ventricle distal to the ligation [28]. The wound was closed using a braided 6 − 0 vicryl suture (J212H, Ethicon). Mice that died within 24 h of surgery were excluded from the analyses (the number of mice that died within the first 24 h in each batch of animal experiments was recorded in Supplementary Materials). Surgery was performed by an operator who was blinded to the identity of the mice and the study protocol.

### Echocardiography

Cardiac function was assessed before and after MI/sham surgery at designated time points using the Vevo 2100 ultrasound system equipped with an MS550D probe (VisualSonics, Fujifilm). Mice were anesthetized with isoflurane inhalation (5% at the induction phase and 1% at the maintenance phase). Mice were placed on a heated platform in a supine position, and the chest fur was removed by depilatory cream. The heart rate of mice was maintained at 500–600 bpm, left ventricular stroke volume (LVSV, µl), left ventricular end-diastolic volume (LVDV, µl), and ejection fraction (EF, %) were measured from a long-axis view by tracing the endocardium. Two independent investigators who were blinded to the identities of mice performed the measurements and analyses using Vevo Lab software (VisualSonics, Fujifilm).

### Histology

The heart tissues were obtained at designated time points after LAD ligation or sham surgery. Hearts were perfused with ice-cold PBS and fixed in 10% formalin at 4 °C overnight. Then the samples were dehydrated and embedded in paraffin 5 μm sections were de-paraffinized and rehydrated. Masson’s trichrome staining was performed according to the manufacturer’s instructions (Sigma-Aldrich). Image J software was employed to quantify the fibrotic area ratio in the left ventricle. To measure cardiomyocyte cross-sectional area (CSA), the heart sections were stained with Alexa Fluor 594 conjugated-WGA (Life Technologies) and the images were observed using a laser confocal microscope (LSM900, ZEISS). The average CSA of randomly selected 50–80 round-shaped cardiomyocytes of each sample was used for analysis, as we previously described [30]. For Mac3 and ROS co-immunofluorescence staining, antigen retrieval with citric acid was performed, followed by blocking with 2.5% goat serum at room temperature for 1 h. Sections were incubated with primary antibodies for nitrotyrosine (Santa Cruz, 1:50 dilution) and Mac3 (rat IgG, Santa Cruz, 1:100 dilution) at 4 °C overnight. Nitrotyrosine was visualized with Alexa Flour 488 Goat Anti-Mouse IgG (invitrogen), and Mac3 with Alexa Flour 594-conjugated anti-rat IgG (invitrogen). Nuclei were stained with DAPI. For Mac3 and IL-1β co-immunofluorescence staining, antigen retrieval with citric acid was performed, followed by blocking with 2.5% goat serum at room temperature for 1 h. Sections were incubated with rabbit anti-IL-1β polyclonal antibody (Bioss) at 1:100 dilution and anti-rat Mac3 antibody (Santa Cruz) at 1:100 dilution at 4 °C overnight. IL-1β was visualized with biotinylated anti-rabbit following fluorescein-labeled streptavidin (Vector Laboratories) and Mac3 with Alexa Flour 594-conjugated anti-rat IgG (Invitrogen). Nuclei were stained with DAPI (Thermo). The images were observed by the laser confocal microscope (LSM900, ZEISS) and analyzed as previously described [28].

### Cell culture

Thioglycollate-elicited macrophages were isolated from the peritoneal cavity of mice 4 days after intraperitoneal injection of 2 ml of 3% thioglycolate broth (BD Difco). After adherence for 6 h, macrophages were washed and cultured in RPMI 1640 medium supplemented with 10% FBS and 1% penicillin/streptomycin. Macrophages were pre-treated with CoQ10 (20 µM, MCE) or vehicle (ethanol, Sigma) for 2 h prior to downstream experiments. Pro-inflammatory activation of macrophages was achieved by treatment with LPS (10ng/ml, Sigma) and IFNγ (2ng/ml, Sigma). For NLRP3 inflammasome activation, LPS/IFNγ-primed macrophages were treated with ATP (5mM, MCE) for 30 min. Macrophages and macrophage culture supernatants were collected for further analysis.

### Intracellular reactive oxygen species (ROS) measurement

The levels of intracellular reactive oxygen species were detected using the Reactive Oxygen Species Assay Kit (DCFH-DA, Beyotime) according to instructions. Briefly, macrophages were stimulated with LPS (1 mg/ml) in the presence of CoQ10 (20µM) or equivalent vehicle (ethanol, sigma) for 6, 12, and 18 h, respectively. Then macrophages were washed and incubated with 10µM DCFH-DA (1:1000) at 37 °C for 30 min in the dark. Cells were harvested after washing with serum-free medium 3 times. The ROS levels were measured using a fluorescence microscope (Olympus IX73), and the intracellular ROS levels were quantified and analyzed by Image J software.

### Flow cytometry and sorting

Hearts were perfused with 20 ml of ice-cold PBS from the apex. The atria and right ventricle were removed, and the left ventricles were minced and digested with collagenase I (450 U/mL), collagenase XI (125 U/mL), DNase I (60 U/mL), and hyalonidase (450 UI/mL) at 37 °C, 900 rpm for 30 min using a ThermoMixer (Eppendorf). Subsequently, the digested suspensions were homogenized through 70 μm cell strainers. Residual red blood cells (RBC) were lysed with RBC lysis buffer (BioLegend) for 20 s at room temperature. The cell surface was stained with antibodies for 20 min at room temperature in the dark. The antibody cocktails include CD45.2-Percp-Cy5.5, Ly6G-PE-Cy7, CD64-APC, Ly6C-FITC, and CCR2-BV421. Downstream intracellular staining was performed using a Foxp3/Transcription factor staining kit (Thermo Fisher). After fixation and permeabilization, cells were further stained with the antibody IL1β-PE (Invitrogen) for 30 min on ice in the dark. Dead cells were excluded with Zombie-Aqua staining (Invitrogen). Cells were defined as previously described [31]. 123 Count Beads were used for counting cell numbers. The antibodies used for flow cytometry analyses are shown in Supplemental Table 2. Flow cytometric data were acquired on a Fortessa flow cytometer (BD Biosciences), and the data were analyzed using FlowJo software. For qPCR analysis of the cardiac CCR2^+^ macrophages (CD45.2^+^Ly6G^−^CD64^+^Ly6C^lo^CCR2^+^), heart digests were prepared as described above, and sorting was performed on the Aria III sorter (BD Biosciences) platform. RNA was extracted using RNeasy micro kit (Qiagen).

### ELISA

BNP in mice serum and secreted IL-1β in macrophage culture supernatants were analyzed using commercial enzyme-linked immunoabsorbent assays (ELISA) according to the manufacturer’s instructions (Protein Tech).

### RNA-Seq

Total RNA from cultured macrophages was extracted using TaKaRa MiniBEST Universal RNA Extraction Kit (TaKaRa). Employing RNA Nano 6000 Assay kit of the Bioanalyzer 2100 system (Agilent Technologies, CA, USA) to assess the RNA integrity. mRNA was purified from total RNA using poly-T oligo-attached magnetic beads and the library fragments were purified with the AMPure XP system (Beckman Coulter, Beverly, USA). The quality of the library was evaluated by the Agilent Bioanalyzer 2100 system. The clustering of the samples was performed on a cBot Cluster Generation System using TruSeq PE Cluster Kit v3-cBot-HS (Illumia) according to the manufacturer’s instructions. Then the library preparations were sequenced on an Illumina Novaseq platform and the quality control was performed by removing reads containing adapter, reads containing ploy-N and low-quality reads from raw data. Hisat2 v2.0.5 was used to build the index of the reference genome and align the paired-end clean reads to the reference genome. FeatureCounts vl.5.0-p3 was employed to count the reads numbers mapped to each gene. And then the FPKM of each gene was calculated based on the length of the gene and the reads count mapped to this gene. The analysis of differential expression genes was performed using the edgeR R package (3.22.5). The p values < 0.05 and |log2fold change (FC)| > 0 were set as the threshold for significantly differential expression in this study. Gene Ontology (GO) enrichment analysis and KEGG enrichment analysis were implemented by the clusterProfiler R package. The terms with p values less than 0.05 were considered significantly enriched by differential expressed genes.

### Gene expression analysis by qRT-PCR

Total RNA from cultured cells was isolated using TaKaRa MiniBEST Universal RNA Extraction Kit (TaKaRa). Total RNA from heart tissues was isolated using QIAzol reagent (Qiagen). RNA was reverse transcribed with PrimeScript RT reagent Kit (TaKaRa) and High-Capacity cDNA Reverse Transcription Kits (Applied Biosystems™). Quantitative real-time PCR was performed with TB Green Premix Ex Taq II (TaKaRa) and QuantiNova SYBR Green PCR Kit (Qiagen) in an Applied Biosystems 7500 Real-Time PCR System. Primers for mouse gene expression analyses are shown in Supplemental Table 3. Results were analyzed with the ∆∆Ct method. 36b4 was used as a reference gene for normalization.

### Western blot

Protein extraction from macrophages was performed using ice-cold lysis buffer supplemented with protease & phosphatase inhibitor cocktail (Thermo Scientific). Equal amounts of protein lysates were resolved by SDS-PAGE. The primary antibodies applied in our study were as follows: rabbit monoclonal anti-NLRP3 (15,101 S, Cell Signaling Technology), rabbit polyclonal anti-IL1β (bs-6319R, Bioss), rabbit monoclonal anti-β-actin (4970T, Cell Signaling Technology). An ImageQuant LAS 4000 imaging system (GE Healthcare) was used for image acquisition and analyses.

### Statistics

Continuous variables were presented as means ± SD or median (interquartile range). Categorical variables were shown as percentages (or frequencies). The Shapiro-Wilk normality test was performed to determine the data distribution. Homogeneity of variance was detected by the F test. Continuous variables with normal distribution were analyzed using two-tailed unpaired Student’s *t-*test for two-group comparisons, and 2-way ANOVA with Tukey’s or Sidak’s multiple comparison tests for multiple-group comparisons. Non-normally distributed data were analyzed using the Mann-Whitney U test for two-group comparisons. The Fisher exact or χ^2^ test was utilized to evaluate two-group differences for categorical variables. The difference in survival rates between the two groups was presented by Kaplan Meier curves and analyzed using the log-rank test. Differences were considered to be statistically significant at *p* < 0.05. All the statistical analyses were performed using GraphPad Prism 8.0.1 (GraphPad Prism Software, La Jolla, California) and Statistical Package for the Social Sciences version 25.0 (SPSS Inc., Chicago, IL, USA).

## Results

### CoQ10 supplementation promotes early recovery of cardiac function in MI patients post PCI

As reported, statins can reduce CoQ10 levels in blood and tissues [22, 32]. To determine the baseline CoQ10 levels in MI patients, we compared the plasma CoQ10 concentrations between age- and gender-pair-matched MI patients and healthy controls. We found that MI patients had significantly lower CoQ10 levels than healthy controls, indicating a deficiency state of plasma CoQ10 in MI patients (Fig. 1B). We further evaluated the effect of CoQ10 supplementation on cardiac recovery of MI in a randomized cohort of 120 MI patients [blank control group (*n* = 59) and CoQ10 treatment group (*n* = 61)]. The baseline characteristics, including sex, age, standard ACS medications (i.e., aspirin, ticagrelor, clopidogrel, statins, β-blockers, ACEI/ARB), Killip classification, GRACE score, triglyceride (TG), Cystatin C (Cys-C) and culprit vessel, etc., were consistent between CoQ10 and control groups (*p* > 0.05) (Supplemental Table 1).

At 1-month and 3-month after PCI during the follow-up period, the increase of ΔEF and ΔFS in MI patients of the CoQ10 group was significantly greater than that in the control group, indicating an accelerated recovery of cardiac function in MI patients of the CoQ10 group (Fig. 1C). Consistently, although there was no statistically significant difference in the change of BNP levels (ΔLnBNP) between the two groups at 1-month after PCI, the decrease of BNP levels in MI patients in the CoQ10 group was significantly greater than that in the control group at 3-month after PCI (Fig. 1D). Collectively, these results suggest that CoQ10 supplementation significantly promotes the early recovery of cardiac function in MI patients after PCI.

### Single CoQ10 treatment alleviates adverse cardiac remodeling in response to experimental MI

**Fig. 2:**
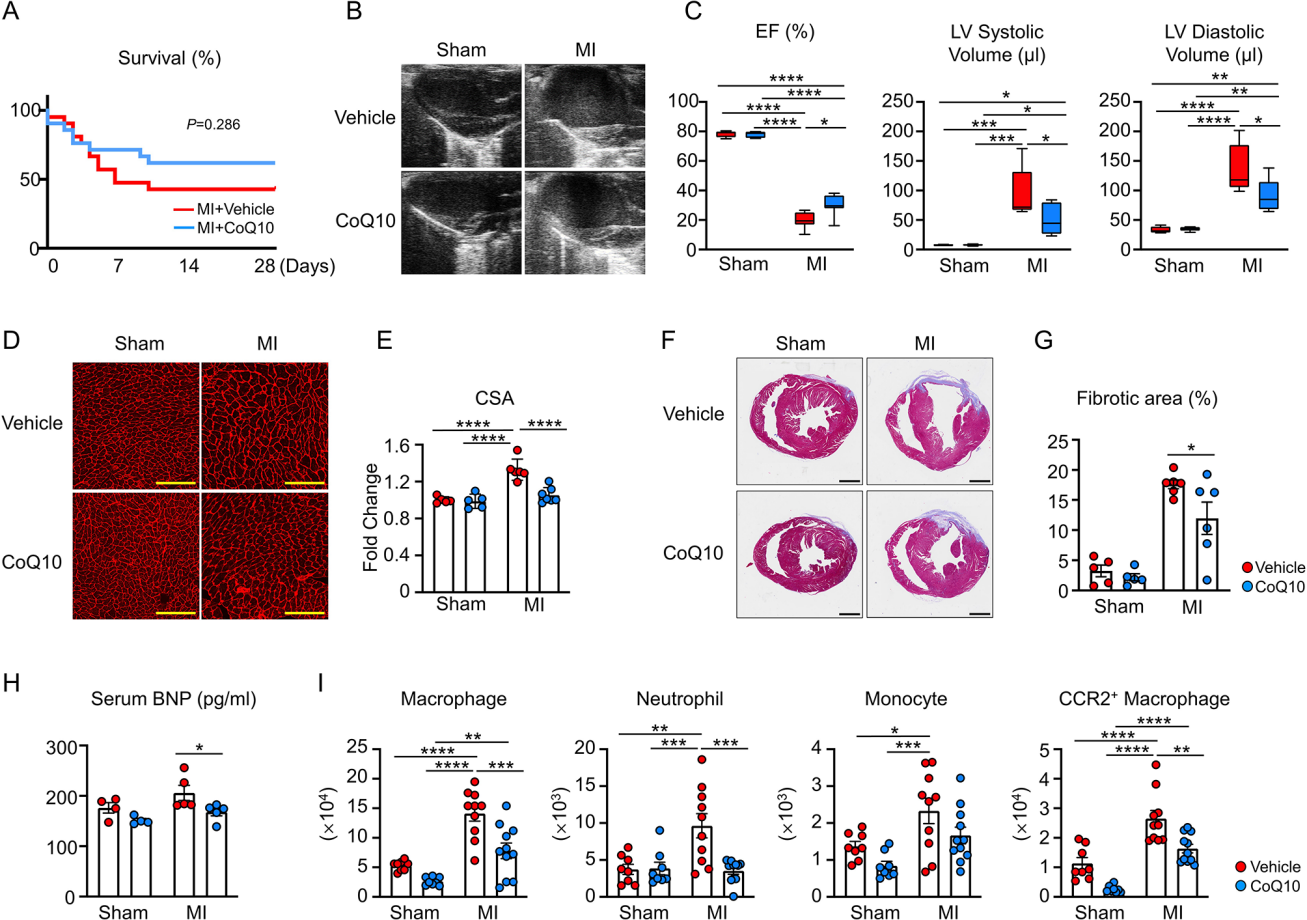
Mice with CoQ10 treatment have a better survival rate and attenuated cardiac remodeling after LAD ligation. **(A)** Kaplan-Meier survival curve of mice administrated with Vehicle or CoQ10 following LAD ligation (Statistical analysis was performed using the log-rank test). **(B)(C)** Representative echocardiographic images and analyses of mice at 28 days after sham or LAD ligation surgeries. Mice were administered with either vehicle or CoQ10 (Statistical analyses were performed using 2-way ANOVA with Tukey’s multiple comparison tests). **(D)(E)** Representative images and analysis of WGA staining of the heart sections from mice of each group at 28 days after sham or LAD ligation surgeries, administrated with vehicle or CoQ10. 200×, scale bars indicate 100 μm (Statistical analysis was performed using 2-way ANOVA with Tukey’s multiple comparison tests). **(F)(G)** Representative images and analysis of Masson’s trichrome staining of the heart tissue sections from mice of each group at 28 days after sham or LAD ligation surgeries, administrated with vehicle or CoQ10. Scale bars indicate 1 mm (Statistical analysis was performed using 2-way ANOVA with Sidak’s multiple comparison tests). **(H)** Serum BNP levels of mice at 28 days after sham or LAD ligation surgeries. Mice were administered with either vehicle or CoQ10 (Statistical analysis was performed using 2-way ANOVA with Sidak’s multiple comparison tests). **(I)** Quantification of flow cytometry analysis of myeloid immune cells in the left ventricles at 28 days after sham/LAD ligation surgeries (Statistical analysis was performed using 2-way ANOVA with Tukey’s multiple comparison tests). Mice were treated with vehicle or CoQ10. MI: myocardial infarction; CoQ10: Coenzyme Q10; EF, ejection fraction; LV: left ventricle; CSA: cross-sectional area of myocyte; BNP, brain natriuretic peptide. *p < 0.05, **p < 0.01, ***p < 0.001, ****p < 0.0001

To explore the protective mechanisms of CoQ10 on ischemic heart disease, we performed LAD ligation to establish the murine model of MI. From 3 days prior to LAD ligation or sham surgery, CoQ10 or vehicle was administered by oral gavage until the end of the study. Compared with MI mice treated with vehicle, the survival rate of MI mice treated with CoQ10 showed an increasing trend throughout the time course (61.90% vs. 42.85%, log-rank test *p* = 0.286; Fig. 2A). Echocardiographic measurements showed that, compared with sham mice, MI mice displayed significantly decreased EF after 28 days of LAD ligation, along with dramatically increased left ventricular (LV) systolic and diastolic volumes. However, CoQ10 treatment significantly alleviated the decrease in EFand relieved the expansion of LV systolic and LV diastolic volumes (Fig. 2B-C), indicating that CoQ10 treatment effectively improved cardiac dysfunction caused by MI. Compared with vehicle-treated MI mice at the terminal 28-day time point, CoQ10-treated MI mice showed significantly reduced cardiomyocyte hypertrophy at the marginal zone (between the infarcted and non-infarcted areas) (Fig. 2D-E). Eearly cardiac fibrosis following LAD ligation was significantly reduced, as shown by histological analyses (Fig. 2F-G), indicating that CoQ10 treatment attenuated the adverse cardiac remodeling after MI. Correspondingly, CoQ10 significantly reduced serum BNP levels in MI mice at 28 days after modeling (Fig. 2H).

As it is increasingly being recognized that the inflammatory response mediated by myeloid cells, especially macrophages, is critical for the progression of cardiac remodeling after myocardial ischemia [7, 33], we tested whether myeloid cells were involved in the cardiac protective effect of CoQ10. Flow cytometry analysis of the infarct area 28 days post-MI showed a significant increase in myeloid cell accumulation in the hearts of vehicle-treated MI mice, notably, the numbers of macrophages and neutrophils were markedly reduced by CoQ10 treatment (Fig. 2I). We further analyzed the impact of CoQ10 on the recruitment of myeloid cells at the inflammatory stage using the hearts isolated 3 days after MI. Although no discernible changes were observed in the absolute numbers of neutrophils and monocytes between MI mice treated with vehicle and those treated with CoQ10, the abundance of macrophages, particularly CCR2^+^ macrophages, was significantly suppressed by CoQ10 treatment (Supplemental Fig. 3).

### CoQ10-treated macrophages exhibit a less inflammatory state and suppressed activation of the NLRP3/IL1β pathway

**Fig. 3:**
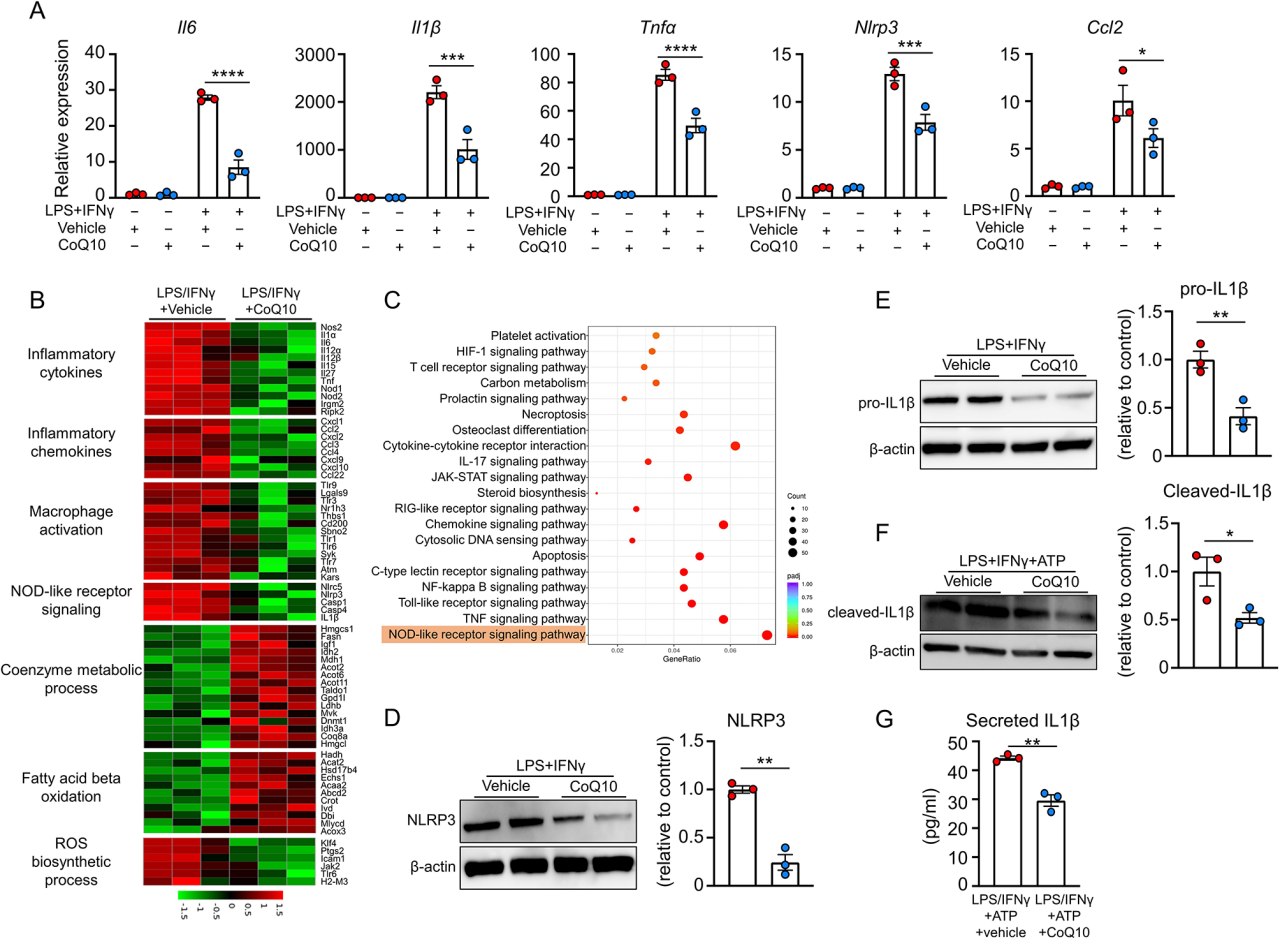
CoQ10 inhibits the ROS generation and activation of NLRP3/IL1β signaling in macrophages. **(A)** Gene expression analysis in peritoneal macrophages with or without 6-hour stimulation of LPS/INFγ, in the presence or absence of CoQ10 (Statistical analysis was performed using 2-way ANOVA with Sidak’s multiple comparison tests). **(B)** Heatmap of RNA script reads of specific genes related to inflammatory cytokines, inflammatory chemokines, macrophage activation, NOD-like receptor signaling, coenzyme metabolic process, fatty acid beta oxidation, ROS biosynthetic process between LPS/IFNγ-primed macrophages treated with or without CoQ10. **(C)** KEGG enrichment analysis of top-ranked pathways of differentially expressed genes between LPS/IFNγ-primed macrophages treated with or without CoQ10. **(D)** Western blot analysis of intracellular NLRP3 in peritoneal macrophages after 6 h of stimulation with LPS/INFγ in the presence of vehicle or CoQ10 (Statistical analysis was performed using a two-tailed unpaired Student’s t-test). Data were normalized to β-actin. **(E)** Western blot analysis of intracellular pro-IL1β in the macrophages after 6 h of stimulation with LPS/INFγ in the presence of vehicle or CoQ10 (Statistical analysis was performed using a two-tailed unpaired Student’s t-test). Data were normalized to β-actin. **(F)** Western blot analysis of intracellular cleaved-IL-1β in the macrophages after 6 h of stimulation with LPS/INFγ combined with a final incubation with 5mM ATP in the presence of vehicle or CoQ10 (Statistical analysis was performed using a two-tailed unpaired Student’s t-test). Data were normalized to β-actin. **(G)** ELISA analysis of secreted IL-1β levels in the supernatant of cultured macrophages after 6 hours’ LPS/INFγ stimulation combined with a final 30 min incubation with 5mM ATP in the presence of vehicle or CoQ10 (Statistical analysis was performed using a two-tailed unpaired Student’s t-test). LPS: lipopolysaccharide; IFNγ: interferon γ; ATP: adenosine triphosphate; CoQ10: Coenzyme Q10; ROS: reactive oxygen species. *p < 0.05, **p < 0.01, ***p < 0.001, ****p < 0.0001

As previously reported, CCR2^+^ macrophages represent the pro-inflammatory macrophage subtype that is closely associated with adverse cardiac remodeling [7, 34, 35] We used primary macrophages to establish the classical polarized macrophage model for ex vivo study to determine the effects of CoQ10 on macrophage inflammatory response. It is well known that the NLRP3 inflammasome is capable of activating caspase-1, which subsequently triggers the release of the inflammatory cytokines (including IL-1β and IL-18), as well as inflammatory cell death (pyroptosis), and promotes the development of the inflammatory cascade response [36]. Upon stimulation with a combination of lipopolysaccharide (LPS) and interferon-γ (IFNγ), cells in the vehicle group displayed significantly upregulated mRNA expression of inflammatory markers of macrophages (such as *iNOS*), as well as various pro-inflammatory cytokines and chemokines [including interleukin-6 (*Il6*), interleukin-1 beta (*Il1β*), tumor necrosis factor-alpha (*Tnfα*), the NOD-like receptor family pyrin domain-containing 3 (*Nlrp3*), *Caspase 1*, interleukin-18 (*Il18*), and chemokine C-C motif ligand 2 (*Ccl2*)]. In comparison, the upregulation of these gene transcripts was significantly attenuated in the CoQ10 group (Fig. 3A, Supplemental Fig. 6), indicating that the macrophage inflammatory state was weakened under CoQ10 treatment.

To explore the molecular mechanisms of the anti-inflammatory effect of CoQ10 on macrophages, we performed next-generation RNA sequencing using RNA from macrophages under the aforementioned conditions. As shown in the manually curated network, many genes associated with inflammation and coenzyme metabolism were differentially expressed between vehicle and CoQ10-treated inflammatory macrophages, (Fig. 3B). Notably, the CoQ10-treated macrophages showed reduced expression of genes associated with inflammatory cytokines, inflammatory chemokines, macrophage activation, and NOD-like receptor (NLR) signaling. Moreover, in CoQ10-treated macrophages, transcripts associated with the coenzyme metabolic process and fatty acid β-oxidation were upregulated, while transcripts associated with the reactive oxygen species (ROS) biosynthetic process were downregulated. ROS has been identified as an important trigger for NLRP3 inflammasome activation in cardiovascular disease [37], which was further confirmed by immunofluorescent staining that showed CoQ10 treatment significantly suppressed the intracellular ROS production in macrophages both in vivo and ex vivo (Supplemental Fig. 4A-D). These results were consistent with the top 10 upregulated and top 10 downregulated biological process and molecular functions revealed by gene ontology (GO) term analysis (Supplemental Fig. 5A-B). Of note, the downregulated CARD domain binding by CoQ10 is closely related to inflammasome formation (Supplemental Fig. 5B). Importantly, as revealed by KEGG pathway enrichment analysis [38–40], the NLR signaling pathway is the predominant pathway mediating the effect of CoQ10 on macrophages under these conditions (Fig. 3C). In addition, compared to the vehicle-treated inflammatory macrophages, CoQ10-treated inflammatory macrophages exhibited lower FPKM values for macrophage inflammatory markers (such as *iNOS*), as well as various pro-inflammatory cytokinesand chemokines (including *Il6*, *Il1β*, *Tnfα*, *Nlrp3*, *Caspase 1, Il18*, and *Ccl2*) (Supplemental Fig. 7).

Being the most extensively studied inflammasome among NLR family members, NLRP3and its downstream cytokine IL1β play a major role in cardiovascular disease by regulating the innate immune response, as we and others reported previously [28, 41, 42]. In light of the downregulated ROS production, *Nlrp3*, and *Il1β* gene transcripts in macrophages by treatment with CoQ10, we explored whether CoQ10 mitigates macrophage-mediated inflammation by inhibiting the activation of the NLRP3/IL1β pathway. To test this possibility, LPS/IFNγ-primed macrophages treated with adenosine triphosphate (ATP) were utilized to establish the two-step signal for inflammasome activation as previously described [43, 44]. Western blot analyses showed that the intracellular expression of NLRP3 and pro-IL1β induced by LPS/IFNγ in macrophages was significantly abolished by CoQ10 treatment (Fig. 3D-E). Furthermore, the intracellular cleaved-IL1β expression induced by a secondary ATP stimulation after LPS/IFNγ was also eliminated by CoQ10, indicating that NLRP3-mediated IL1β processing was attenuated under CoQ10 treatment (Fig. 3F). Consistently, the secreted extracellular IL1β expression was significantly diminished by CoQ10 treatment, as revealed by the enzyme-linked immunosorbent assay (ELISA) (Fig. 3G). Overall, these results support the hypothesis that CoQ10 inhibits the activation of the NLRP3/IL1β pathway in macrophages.

### CoQ10 mitigates post-infarction cardiac inflammation associated with IL1β signaling

**Fig. 4:**
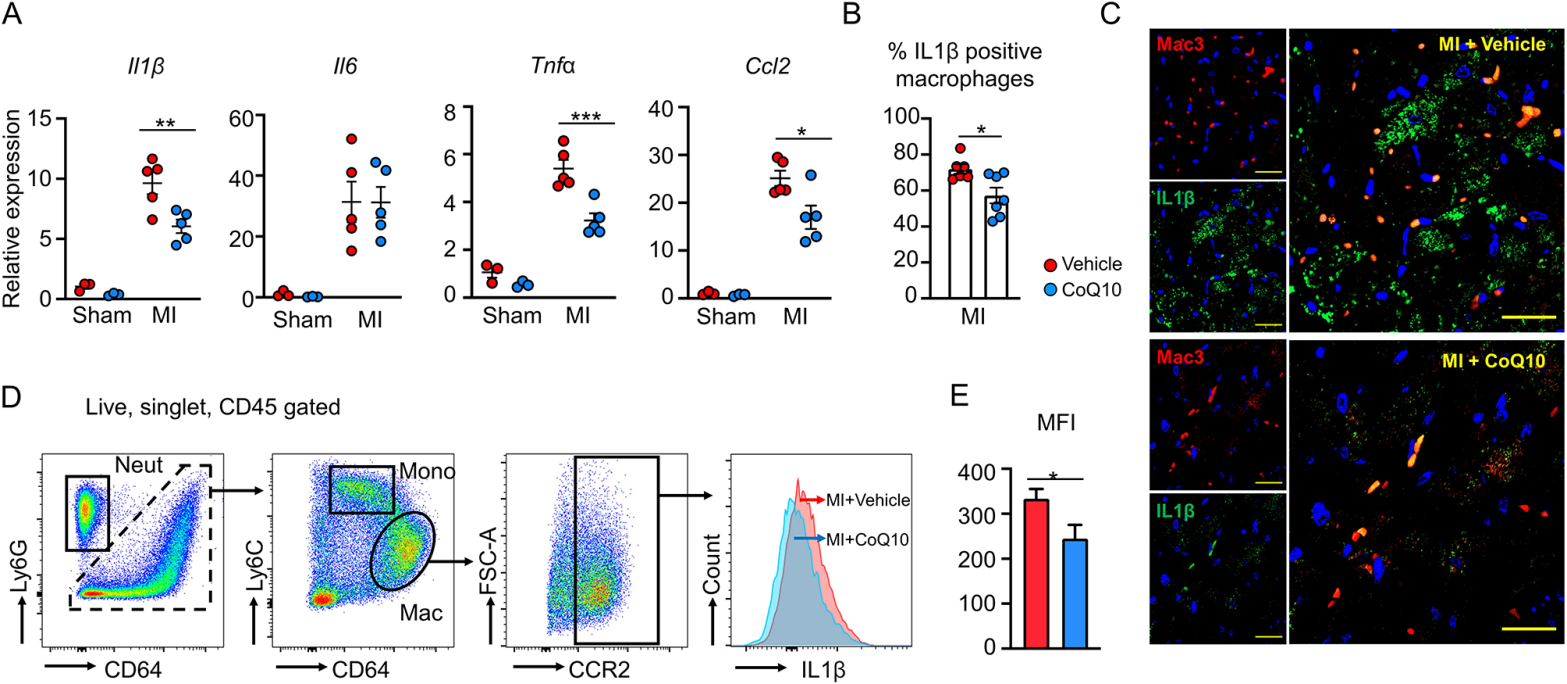
IL1β production-related post-infarction cardiac inflammation was suppressed by CoQ10 treatment. **(A)** Analysis of gene expression in the infarct myocardium from each group of mice at 3 days after sham/LAD-ligation surgeries (Statistical analysis was performed using 2-way ANOVA with Sidak’s multiple comparison tests). Mice were treated with either vehicle or CoQ10. **(B)(C)** Representative images and analysis of the IL1β immunofluorescence staining in the Mac3-positive macrophages from mice treated with vehicle or CoQ10 at 28 days after LAD ligation (Statistical analysis was performed using a two-tailed unpaired Student’s t-test). 630×. Scale bars indicate 20 μm. **(D)** Representative flow cytometry analysis of left ventricles after sham/LAD ligation surgeries. Cells were defined as neutrophils (Neut, CD45.2^+^CD64^−^Ly6G^+^), monocytes (Mono, CD45.2^+^Ly6G^−^CD64^int^Ly6C^hi^), macrophages (Mac, CD45.2^+^Ly6G^−^CD64^+^Ly6C^lo^), or CCR2^+^ macrophages (CCR2^+^Mac, CD45.2^+^Ly6G^−^CD64^+^Ly6C^lo^CCR2^+^). (E) Quantification of the mean fluorescence intensity (MFI) of IL-1β in the CCR2^+^ macrophages of mice 3 days after LAD ligation, either treated with vehicle or CoQ10 (Statistical analysis was performed using a two-tailed unpaired Student’s t-test). MI: myocardial infarction; CoQ10: Coenzyme Q10; MFI: mean fluorescent intensity. *p < 0.05, **p < 0.01, ***p < 0.001

Having demonstrated the role of CoQ10 on the activation of the NLRP3/IL1β pathway and downstream inflammatory response in macrophages, we next explored the impact of CoQ10 on macrophage-mediated inflammation in the context of myocardial infarction. Gene expression analysis was performed to check the inflammatory status of the heart using the tissues from the infarct myocardium at 3 days and 28 days post-LAD ligation. The infarct myocardium of post-LAD ligation in vehicle-treated MI mice displayed significantly upregulated mRNA expression of *Il1β*, *Il6*, *Tnfα*, *Nlrp3, Caspase1, Il18, iNOS*, and *Ccl2* compared with sham mice, indicating the enhanced inflammation in the infarct area induced by LAD ligation. However, consistent with our observations in the primary macrophages (Fig. 3A), CoQ10 treatment significantly downregulated the gene transcripts of *Il1β*, *Tnfα*, and *Ccl2*, whereas no difference in *Il6, Nlrp3, or iNOS* was observed between vehicle and CoQ10-treated MI mice at 3 days after LAD ligation (Fig. 4A, Supplemental Fig. 8). When compared to the infarcted myocardium of vehicle-treated MI mice at 28 days after LAD ligation, similar results were observed: CoQ10 treatment effectively reduced the gene transcripts of pro-inflammatory cytokines (including *Il6, Tnfα, Nlrp3, and Caspase1*) in the CoQ10-treated MI mice (Supplemental Fig. 9). Furthermore, using flow cytometry sorting technology, CCR2^+^ macrophages were obtained from the infarct myocardium of MI mice (at 3 days following LAD ligation), subsequently, qPCR was performed to assess these macrophages’ inflammatory status (Supplemental Fig. 10A). As results have shown, compared with vehicle-treated MI mice, CoQ10 treatment significantly suppressed the gene expression of pro-inflammatory cytokines (including *Il1β, Il6, Tnfα, Nlrp3, and Il18*) in CCR2^+^ macrophages of infarct myocardium (Supplemental Fig. 10B). These data suggest a lower inflammatory status in the infarct myocardium (including CCR2^+^ macrophages from the infarct myocardium) under CoQ10 treatment.

Immunofluorescent staining of tissues from infarct myocardium at 28 days post-LAD ligation also revealed that CoQ10-treated MI mice had significantly fewer IL1β-positive macrophages compared with those in vehicle-treated MI mice (Fig. 4B-C). In support of this finding, flow cytometry analysis showed that CCR2^+^ macrophages from the infarct myocardium of CoQ10-treated MI mice at 3 days post-LAD ligation expressed significantly lower protein levels of IL1β compared to those of vehicle-treated MI mice (Fig. 4D-E). Overall, these data support the central role of attenuated IL1β signaling in CoQ10-mediated inflammation reduction after myocardial infarction.

## Discussion

Extensive studies have revealed that persistent inflammation after intensive lipid therapy is the main residual risk for major adverse cardiovascular events in Atherosclerotic Cardiovascular Disease (ASCVD) patients [45]. Three prominent clinical trials published in recent years (CANTOS, COLCOT, and LodoCo2) demonstrated that targeted inhibition of the NLRP/IL1β inflammatory axis can significantly improve the prognosis of ASCVD patients, suggesting NLRP/IL1β as an ideal target for anti-inflammatory therapy of ASCVD [11–13]. Being a naturally existing compound in humans and animals, CoQ10 has been reported in many studies to play an important role in cellular ATP production and scavenging oxygen-free radicals [17–19]. Supplementation with CoQ10 has been reported to improve the long-term prognosis of patients with MI or HF with high safety [46, 47]. More recently, a systematic review and meta-analysis of randomized controlled intervention trials demonstrated moderate-to-high-quality evidence that CoQ10 supplementation significantly decreased all-cause mortality events [25]. However, it remains unclear whether CoQ10 can alleviate MI injury and prevent its progression to HF by regulating the early inflammatory stage. In this study, we found that MI patients showed a deficiency state of plasma CoQ10 at baseline, and early supplementation with CoQ10 after PCI promoted the recovery of cardiac function in MI patients. In animal models, single use of CoQ10 treatment significantly reduced mortality after MI surgery and significantly reduced cardiac function deterioration and adverse cardiac remodeling induced by MI. The anti-inflammatory effect of CoQ10 may be attributed to significantly mitigating NLRP3/IL1β pathway-mediated inflammation in macrophages and suppressing the recruitment of inflammatory macrophages to the ischemic myocardium post-MI.

CoQ10 is a coenzyme of the mitochondrial complex with a role in promoting cellular energy generation and antioxidant activity. As such, the abundance of CoQ10 is high in tissues with high metabolic demands, such as the heart and kidney. Aging and some drugs that affect the synthesis of CoQ10 (such as statins) have been reported to decrease the levels of CoQ10 in blood and tissues [32, 48]. Previous reports demonstrated that in patients with cardiomyopathy, the severity of cardiac function is inversely correlated with the level of CoQ10 in tissues and blood [49]. In our study, we found that MI patients displayed a deficiency state of plasma CoQ10 in comparison with healthy controls with age and gender pair-matched, this may be related to the routine statin administration of ASCVD patients.

Research on CoQ10 in cardiovascular diseases mainly focuses on HF to date. Clinical studies and meta-analyses mostly agree that CoQ10 has a protective effect on improving the long-term prognosis of HF and reducing adverse cardiovascular events. Especially in the most famous Q-SYMBIO study to date, the authors didn’t find statistically significant differences in the primary short-term endpoints (including the 6-minute walk test, NYHA functional class, and NT-pro BNP) between CoQ10 and placebo-treated HF patients at 4-month follow-up. But CoQ10 significantly reduced the risk of adverse cardiovascular events in HF patients at 2 years of follow-up. Notably, a trend toward improved LVEF was only detected in CoQ10-treated HF patients with baseline LVEF greater than 30%but not in those with LVEF lower than 30% [23, 26]. In the current study, the baseline LVEF of MI patients in both groups was higher than 45%. Of note, we found that the beneficial changes of LVEF and LVFS at 1- month and 3-month follow-up, as well as the reductions in changes of LnBNP at 3-month of follow-up, were all significantly greater in CoQ10-treated MI patients, which suggests that early supplementation with CoQ10 after PCI could effectively promote the recovery of cardiac function and prevent its progression to HF. In animal models, we used LAD ligation to establish an experimental MI model, and we found that in the absence of baseline CoQ10 deficiency and the use of statins that affect CoQ10 levels, the single use of CoQ10 treatment showed a trend of improved survival rate, significantly reduced BNP levels, improved cardiac dysfunction, and adverse cardiac remodeling induced by MI, further supporting that early supplementation with CoQ10 is beneficial for improving MI prognosis.

As reported, the tissue infiltration of innate immune cells (including macrophages, monocytes, neutrophils, etc.) and the inflammatory microenvironment formed after ischemic injury are important in regulating MI prognosis [6, 50]. Notably, it has been appreciated that the source and function of macrophages in the heart are heterogeneous, including yolk-sac-derived reparative resident macrophages and bone marrow-derived pro-inflammatory macrophages [34, 51, 52]. CCR2^+^ macrophage represents the pro-inflammatory subtype that highly expresses inflammatory cytokines and chemokines, playing a critical role in persistent cardiac inflammation [7]. In this study, we found that the accumulation of macrophages (including CCR2^+^ macrophages) and neutrophils was significantly inhibited by CoQ10 treatment at the remodeling stage. However, at the earlier inflammatory stage post-infarction, monocytes, macrophages, and neutrophils all largely expanded in the infarct myocardium, while CoQ10 treatment mainly inhibited the recruitment of macrophages, particularly CCR2^+^ macrophages. These data suggest that the dampened inflammation mediated by CCR2^+^ macrophages may be the main mechanism of CoQ10 protection in this model.

In order to understand how CoQ10 regulates macrophage-mediated inflammation, we established the classic polarized macrophage model and performed gene expression analysis and unbiased RNA sequencing analysis. Remarkably, CoQ10 treatment led to a significant downregulation of genes associated with the innate immune response, including inflammatory cytokines, chemokines, macrophage activation, and NLR signaling components (*Nlrp3*, *Caspase1*, *Il1β, Il18*, etc.), as well as genes related to ROS generation. The up-regulated genes upon CoQ10 treatment were mainly associated with coenzyme metabolism, fatty acid β-oxidation, and oxidoreductase activities, which have been previously reported to improve mitochondrial function and reduce ROS generation [53]. Consistently, this result was further confirmed by immunofluorescent staining, which showed that CoQ10 treatment inhibited ROS production in inflammatory macrophages and reduced ROS abundance in macrophages of infarct myocardium. Of note, ROS is a trigger for the activation of the NLRP3 inflammasome and the secretion of the downstream cytokine IL1β [54]. Further, the KEGG analysis showed that NLR signaling is the predominant pathway that mediated the anti-inflammatory effect of CoQ10 in macrophages. Taken together, these results demonstrated that CoQ10 might suppress the activation of NLRP3/IL1β signaling by inhibiting excessive ROS production.

As previously reported, IL1β is synthesized in an inactive form (pro-IL1β), and its secretion requires NLRP3-mediated cleavage, while the activation of the NLRP3 inflammasome requires a typical two-step stimulation [55, 56]. In the current study, LPS and IFNγ were used for the priming signal to promote the expression of pro-IL1β and NLRP3, and further, ATP treatment was used as the activation signal to activate the assembly of the NLRP3 inflammasome and promote the secretion of mature IL1β. Importantly, our western blot analysis confirmed that CoQ10 treatment significantly inhibited the activation of the NLRP3 inflammasome, resulting in attenuated synthesis and secretion of IL1β.

Notably, the chemokine *Ccl2* is the ligand of CCR2, and the recruitment of CCR2 macrophages is regulated by *Ccl2* levels [57]. Consistent with our finding that CoQ10 treatment suppressed the recruitment of CCR2^+^ macrophages to ischemic myocardium, we detected a significantly lower *Ccl2* mRNA expression in the CoQ10-treated infarct tissues and macrophages. Additionally, as the molecular reactor of pro-inflammatory macrophages, IL1β signaling was found to be attenuated under CoQ10 treatment not only in polarized macrophages but also in whole heart and cardiac macrophages in the context of MI. Specifically, CoQ10 treatment resulted in downregulated IL1β expressions in infarct myocardium and CCR2^+^ macrophages at the early inflammatory stage, as well as reduced IL1β expression in cardiac macrophages at the remodeling stage post-MI. These results indicate that CoQ10 treatment continuously inhibited the expression of the pro-inflammatory cytokine IL1β in cardiac macrophages at all stages after MI. In summary, these data strongly support that the anti-inflammatory effect of CoQ10 after MI is associated with the attenuation of IL1β signaling in inflammatory macrophages.

### Limitations

We acknowledge that limitations exist in the current study. First, we didn’t explore if there is a dose-dependent effect of CoQ10 on cardiac phenotype due to the limited cohort size. Although the dose of CoQ10 used in clinical trials is mostly between 100 and 400 mg/day, in the current study, we used a drug form of CoQ10 approved by the National Medical Products Administration of China at 30 mg/day instead of a nutritional supplement. And the study has proven that this drug dose is effective. In addition, it is known that the level of CoQ10 in heart tissue is significantly higher than that in blood, and the effect of statins on decreasing the level of CoQ10 is more significant in tissues than that in blood. However, due to ethical constraints, this study only detected the plasma level of CoQ10, and we still found the plasma deficiency state of CoQ10 in MI patients. Second, a comprehensive assessment of the bioavailability of the CoQ10 we used was not conducted in our study. Finally, the KEGG analysis of RNA sequencing showed that several signaling pathways were involved in the effect of CoQ10. Thus, we cannot rule out the possibility that other inflammatory pathways or cytokines might be targets of CoQ10 as well, which needs to be verified in future studies. However, the results of the current study strongly support that the effect of CoQ10 on MI outcome is associated with attenuated NLRP3/IL1β signaling in cardiac macrophages.

## Conclusion

In summary, we demonstrated that Coenzyme Q10 treatment can effectively promote the recovery of cardiac function after myocardial infarction and prevent its progression to heart failure, partially through inhibiting NLRP3/IL1β pathway-mediated inflammation in macrophages.

## Electronic supplementary material

Below is the link to the electronic supplementary material.

Supplementary Material 1

## Data Availability

Relevant data have been presented in the main manuscript. The RNA-Seq datasets generated and analyzed during the current study are available in the [BioProject database] repository [http://www.ncbi.nlm.nih.gov/bioproject/979940; BioProject ID: PRJNA979940]. Additional datasets used and/or analyzed during the current study are available from the corresponding author upon reasonable request.

## Abbreviations

ASCVD: atherosclerotic cardiovascular disease
ATP: adenosine triphosphate
CoQ10: coenzyme Q10
CCR2: chemokine C-X-C motif receptor
CCL2: chemokine (C-C motif) ligand 2
EF: ejection fraction
FS: fractional shortening
HF: heart failure
IL1β: interleukin-1 β
IL6: interleukin 6
IFNγ: interferon γ
LV: left ventricular
LPS: lipopolysaccharide
LAD: left anterior descending coronary artery
MI: myocardial infarction
NLRP3: NOD-like receptor family pyrin domain-containing 3
PCI: percutaneous coronary intervention
ROS: reactive oxygen species
TNFα: tumor necrosis factor α
