# Correction: Coenzyme Q10 mitigates macrophage mediated inflammation in heart following myocardial infarction via the NLRP3/IL1β pathway

**DOI:** 10.1186/s12872-024-04195-1

**Published:** 2024-10-05

**Authors:** Wenxu Pan, Guiquan Zhou, Meiling Hu, Gaoshan Li, Mingle Zhang, Hao Yang, Kunyan Li, Jingwei Li, Ting Liu, Ying Wang, Jun Jin

**Affiliations:** Department of Cardiology, The Second Affiliated Hospital of Army Medical University, Chongqing, China

**BMC Cardiovascular Disorders (2024) 24:76**

10.1186/s12872-024-03729-x

Following publication of the original article [1], the grant number relating to National Natural Science Foundation of China was incorrectly given as 82100218 and should have been 82100288.

The original article has been corrected.
