# Supplementary material for: Coenzyme Q10 mitigates macrophage mediated inflammation in heart following myocardial infarction via the NLRP3/IL1β pathway
Source: BMC Cardiovasc Disord. 2024 Jan 28;24:76. doi: 10.1186/s12872-024-03729-x (PMC10822151; doi:10.1186/s12872-024-03729-x)
Supplement: Supplementary file 1 — Supplementary Material 1 [file 12872_2024_3729_MOESM1_ESM.docx]

**Supplementary Material**

**Supplemental Figures**


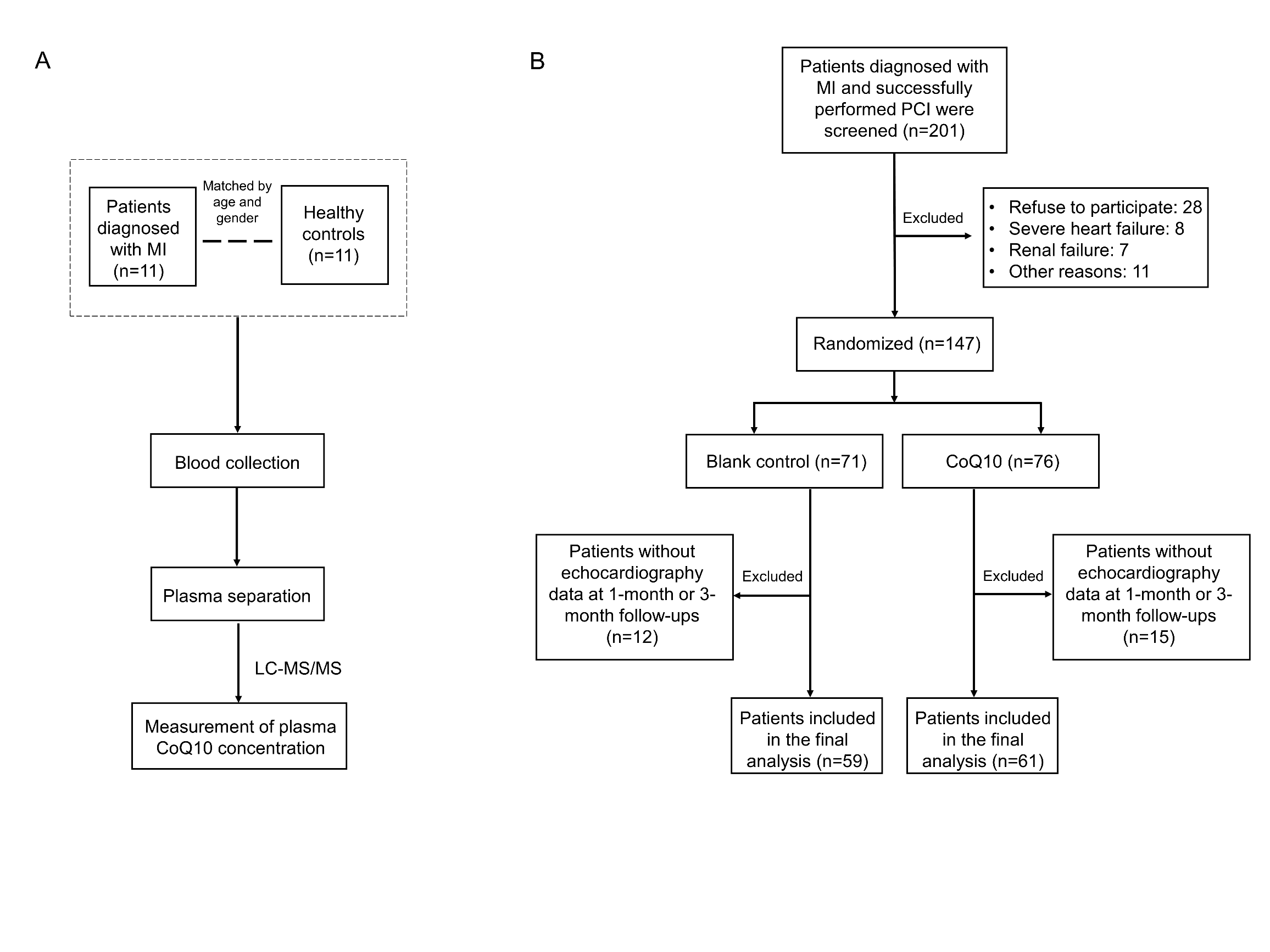


Supplemental Figure 1. Flowchart of the clinical study. (A) To evaluate the baseline plasma concentration of CoQ10 between healthy controls and MI patients. (B) Our ongoing clinical trial (To investigate the impact of CoQ10 supplementation on the recovery of cardiac function in MI patients). MI, myocardial infarction; PCI, percutaneous coronary intervention; LC-MS/MS, liquid chromatographic-tandem mass spectra-metric; CoQ10, Coenzyme Q10.

**
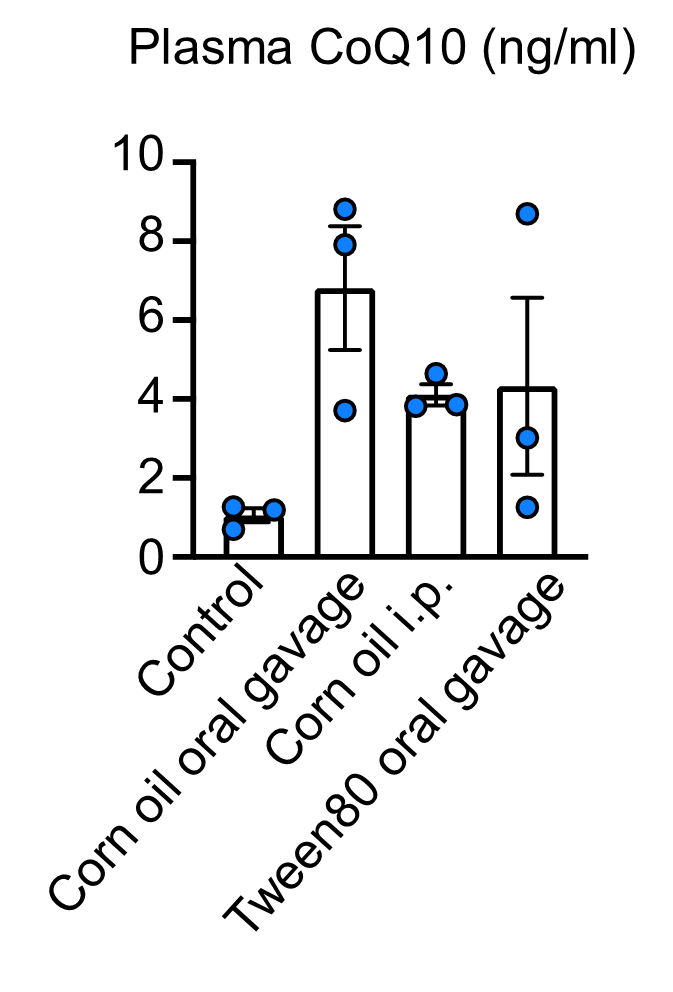
**

Supplemental Figure 2. Plasma coenzyme Q10 concentration was achieved by various administration methods in mice. CoQ10 was administered in 3 different methods, including CoQ10 dissolved in corn oil + oral gavage, CoQ10 dissolved in corn oil + peritoneal injection, and CoQ10 dissolved in Tween80 + oral gavage. Mice without treatment were used as controls. Plasma coenzyme Q10 concentrations of these mice were analyzed by LC-MS/MS at 3 days after administration. CoQ10, Coenzyme Q10; i.p., intraperitoneal


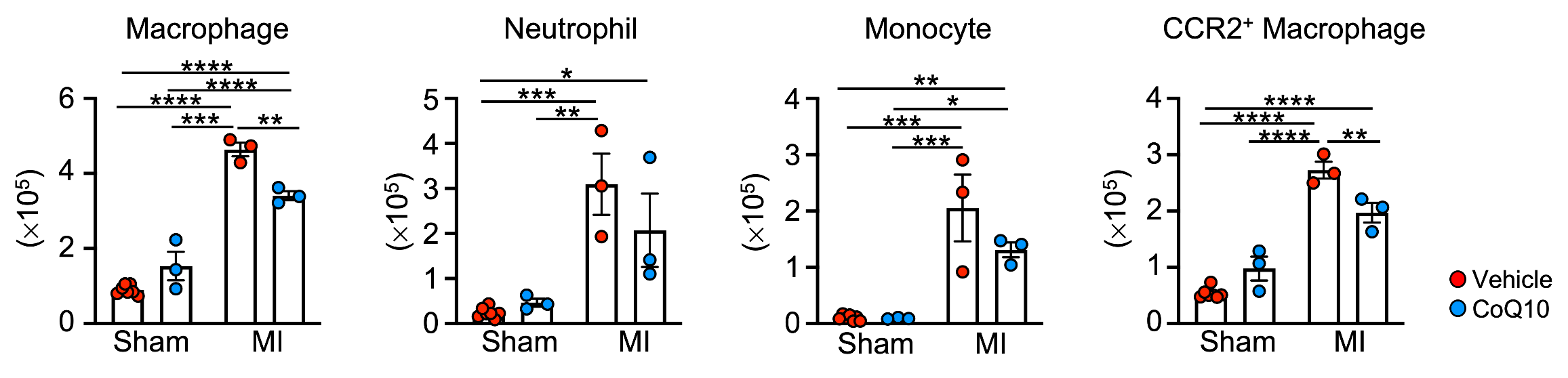


Supplemental Figure 3. Coenzyme Q10 administration inhibits the recruitment of pro-inflammatory macrophages into infarct myocardium (Statistical analyses were performed using 2-way ANOVA with Tukey’s multiple comparison tests). Quantification of flow cytometry analysis of myeloid immune cells in the left ventricles at 3 days after sham/LAD ligation surgeries. Mice were treated with vehicle or CoQ10. MI, myocardial infarction; CoQ10, Coenzyme Q10. **p*<0.05, ***p*<0.01, ****p*<0.001, *****p*<0.0001.


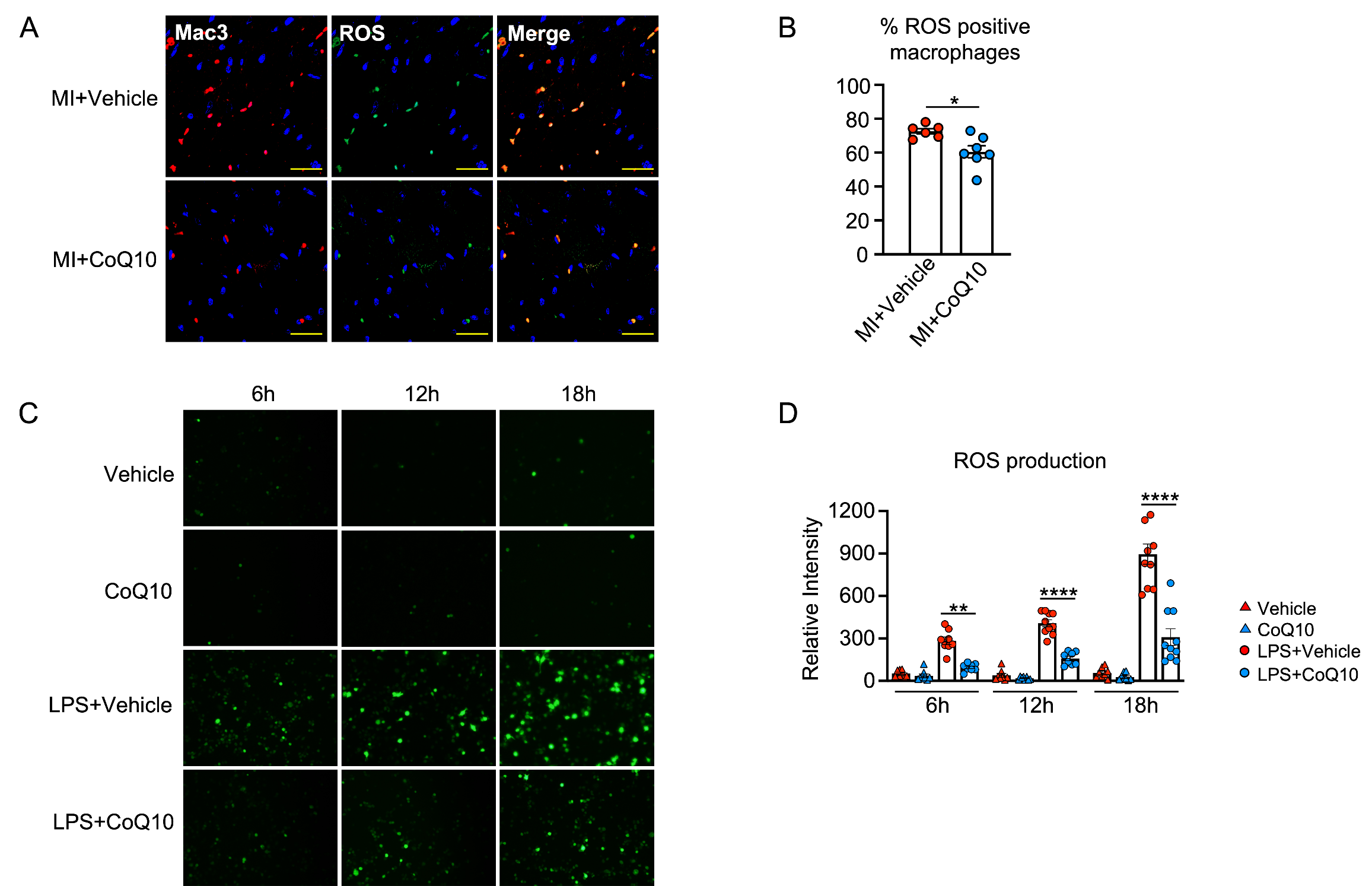


Supplemental Figure 4. Coenzyme Q10 treatment suppresses the production of intracellular ROS in macrophages. (A)(B) Representative images and quantification analysis of ROS immunofluorescence staining in the Mac3-positive macrophages from mice treated with vehicle or CoQ10 for 28 days after LAD ligation (Statistical analysis was performed using a two-tailed unpaired Student’s t-test). 630×. Scale bars indicate 20μm. (C)(D) Representative images and quantification analysis of intracellular ROS production in peritoneal macrophages treated with vehicle or CoQ10, in the absence or presence of LPS stimulation for 6, 12, and 18 hours, respectively (Statistical analysis was performed using 2-way ANOVA with Tukey’s multiple comparison tests). 200×. MI, myocardial infarction; ROS, reactive oxygen species; LPS, lipopolysaccharide; CoQ10, Coenzyme Q10. **p*<0.05, ***p*<0.01, *****p*<0.0001.


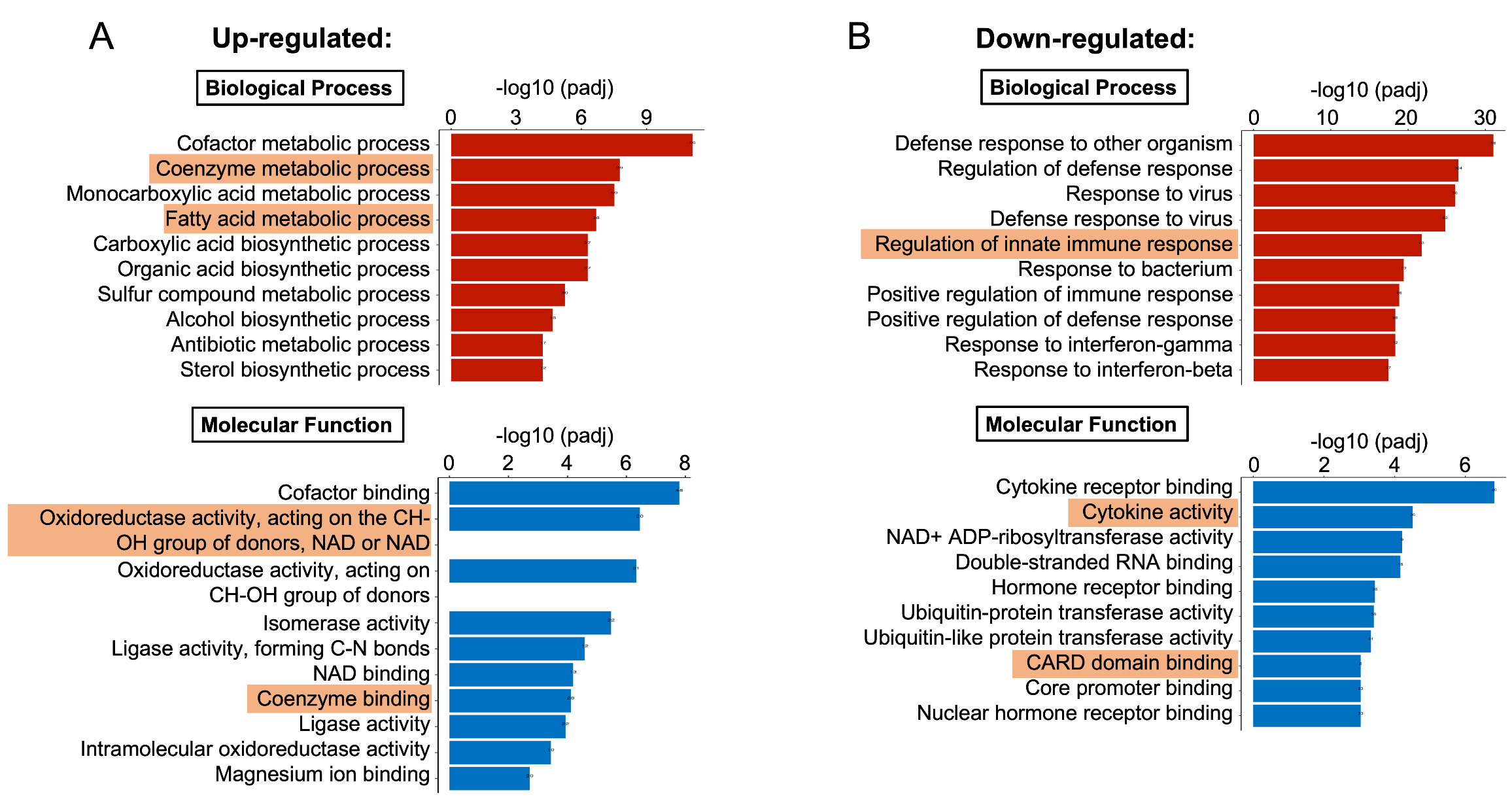


Supplemental Figure 5. Transcriptome analysis reveals the anti-inflammatory signature of Coenzyme Q10 in macrophages. (A) Top-ranked annotation categories of biological process and molecular function for genes up-regulated by CoQ10 administration in inflammatory macrophages. (B) Top-ranked annotation categories of biological process and molecular function for genes down-regulated by CoQ10 administration in inflammatory macrophages. padj, adjusted *p* value.


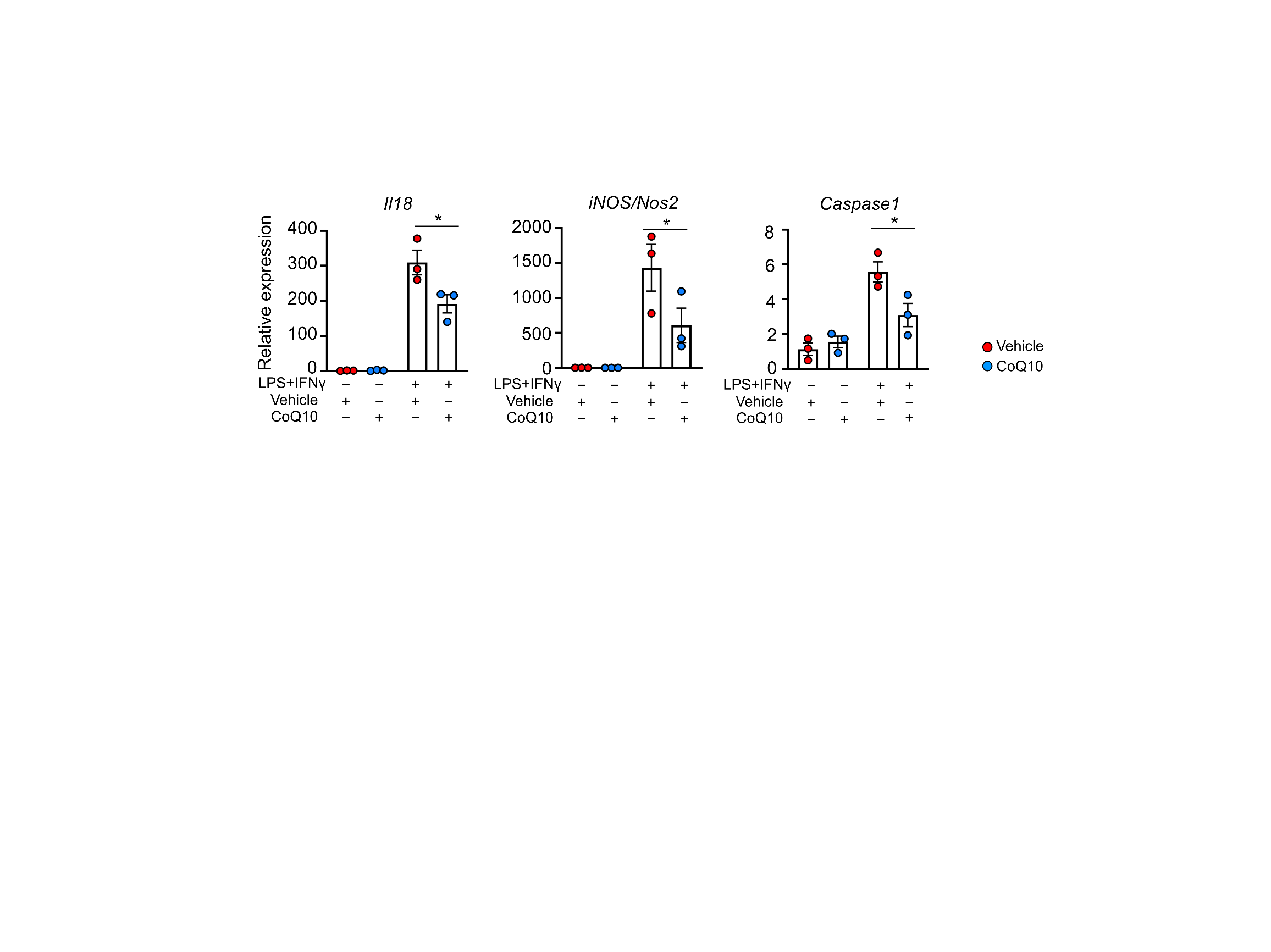


Supplemental Figure 6. Gene expression analysis in peritoneal macrophages with or without 6-hour stimulation of LPS/INFγ, in the presence or absence of CoQ10 (Statistical analysis was performed using 2-way ANOVA with Sidak’s multiple comparison tests). LPS, lipopolysaccharide; IFNγ, interferon γ; CoQ10, Coenzyme Q10. **p*<0.05.


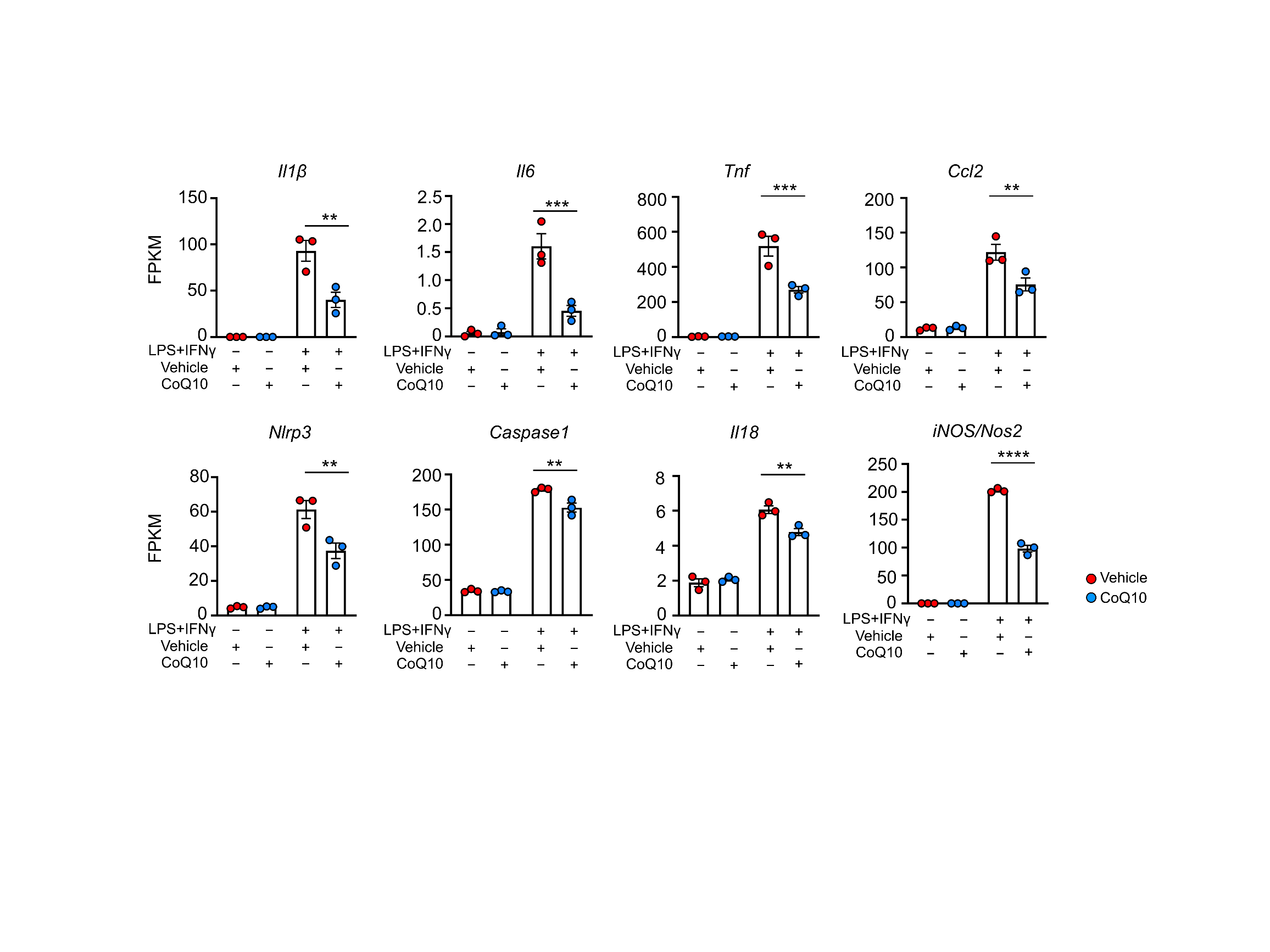


Supplemental Figure 7. FPKM values between peritoneal macrophages with or without stimulation of LPS/INFγ and treated with or without CoQ10 (Statistical analysis was performed using 2-way ANOVA with Sidak’s multiple comparison tests). LPS, lipopolysaccharide; IFNγ, interferon γ; FPKM, fragments per kilobase of exon per million fragments mapped. CoQ10, Coenzyme Q10; ***p*<0.01, ****p*<0.001, *****p*<0.0001.


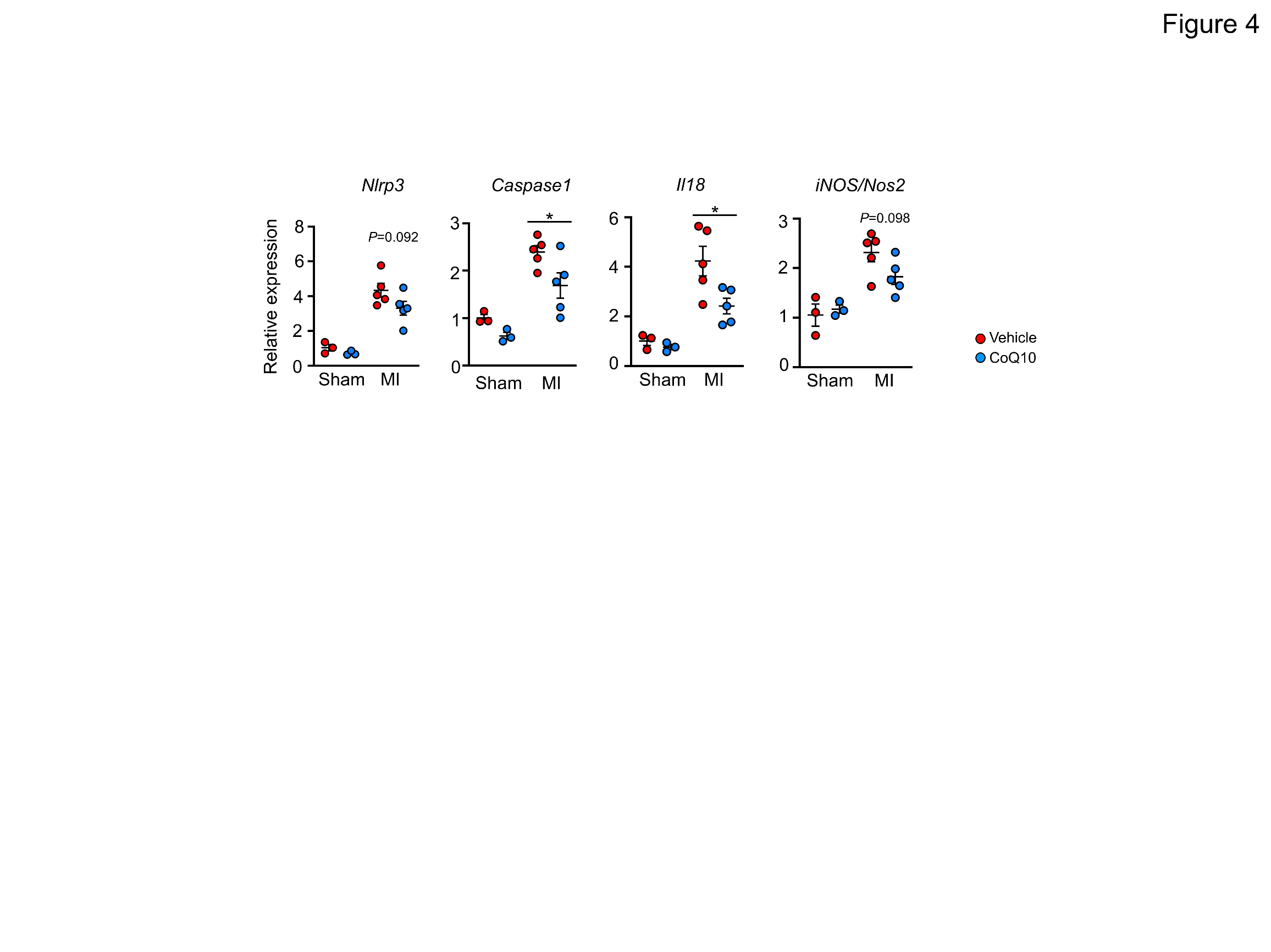


Supplemental Figure 8. Analysis of gene expression in the infarct myocardium from each group of mice at 3 days after sham/LAD-ligation surgeries (Statistical analysis was performed using 2-way ANOVA with Sidak’s multiple comparison tests). Mice were treated with either vehicle or CoQ10. MI, myocardial infarction; CoQ10, Coenzyme Q10. **p*<0.05.


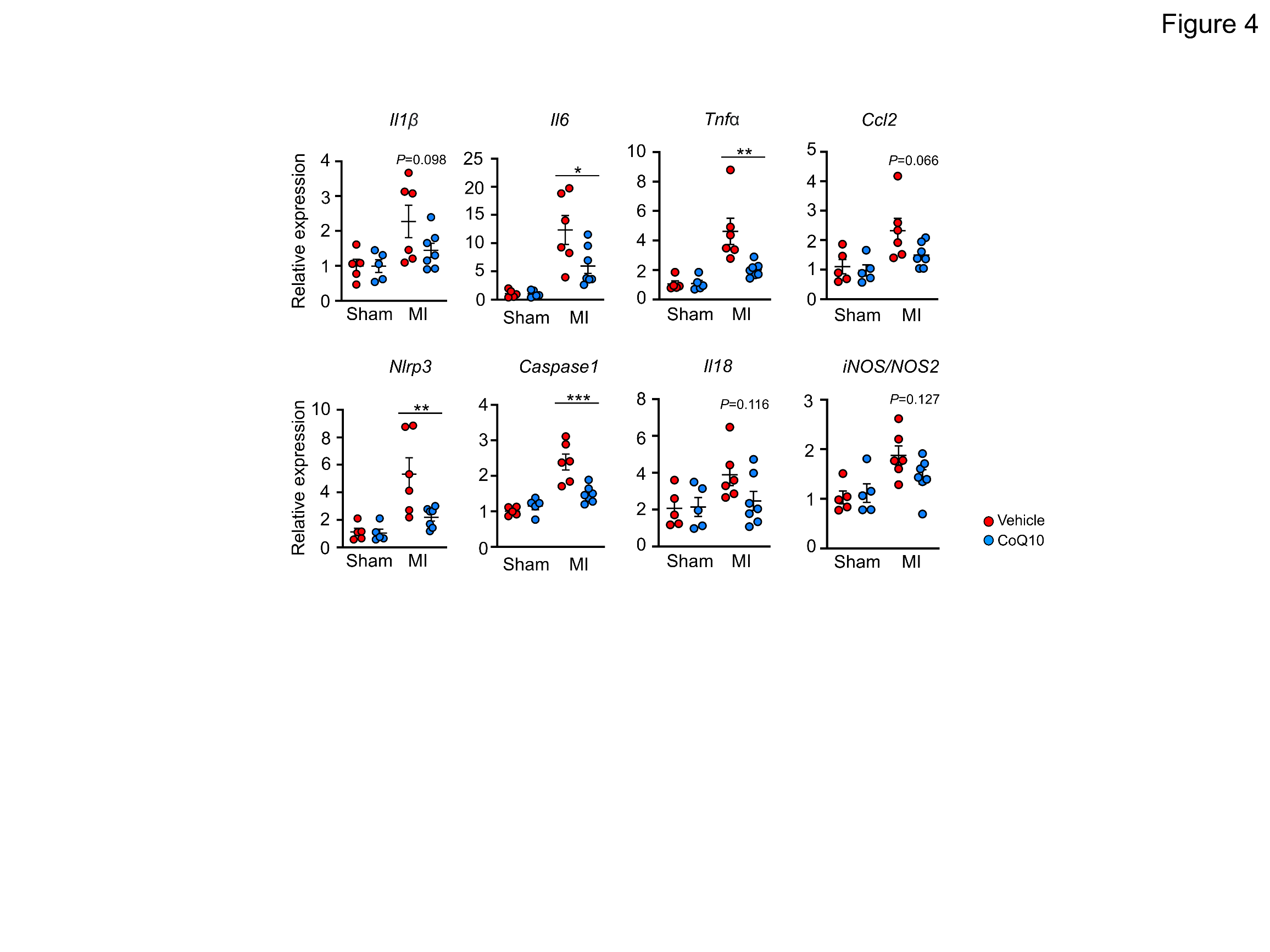


Supplemental Figure 9. Analysis of gene expression in the infarct myocardium from each group of mice at 28 days after sham/LAD-ligation surgeries (Statistical analysis was performed using 2-way ANOVA with Sidak’s multiple comparison tests). Mice were treated with either vehicle or CoQ10. MI, myocardial infarction; CoQ10, Coenzyme Q10. **p*<0.05, ***p*<0.01, ****p*<0.001.


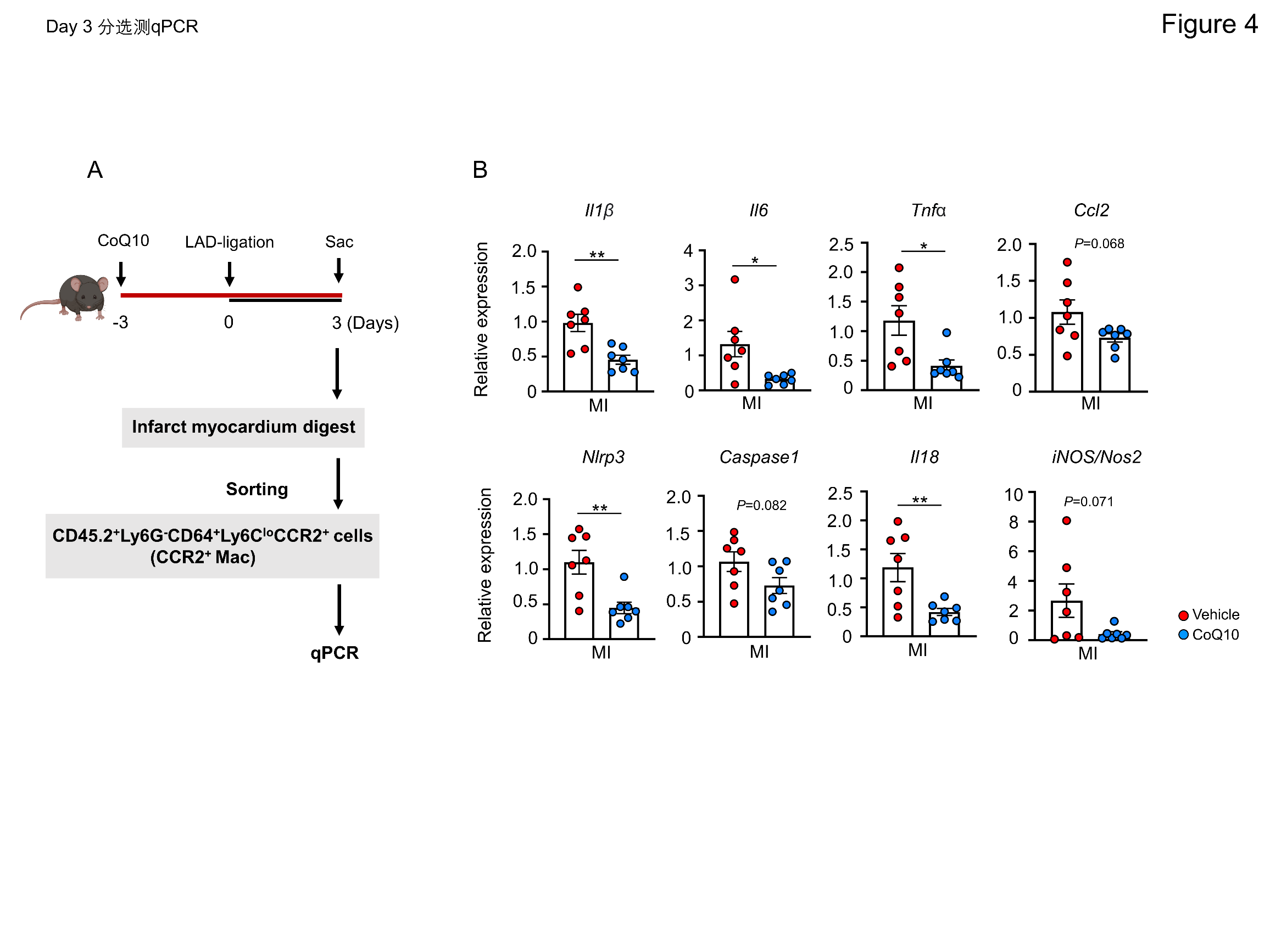


Supplemental Figure 10. CoQ10 inhibits the inflammatory state of CCR2^+^ macrophages recruited in infarct myocardium. (A) The strategy for sorting CCR2^+^ macrophages from the infarct myocardium at 3 days after LAD ligation. (B) Gene expression analysis of CCR2^+^ macrophages sorted from the infarct myocardium at 3 days following LAD ligation (Statistical analysis was performed using a two-tailed unpaired Student’s t-test). Mac, macrophages; LAD, left anterior descending coronary artery; Sac, sacrifice; Mac, macrophage; CoQ10, Coenzyme Q10. **p*<0.05, ***p*<0.01.


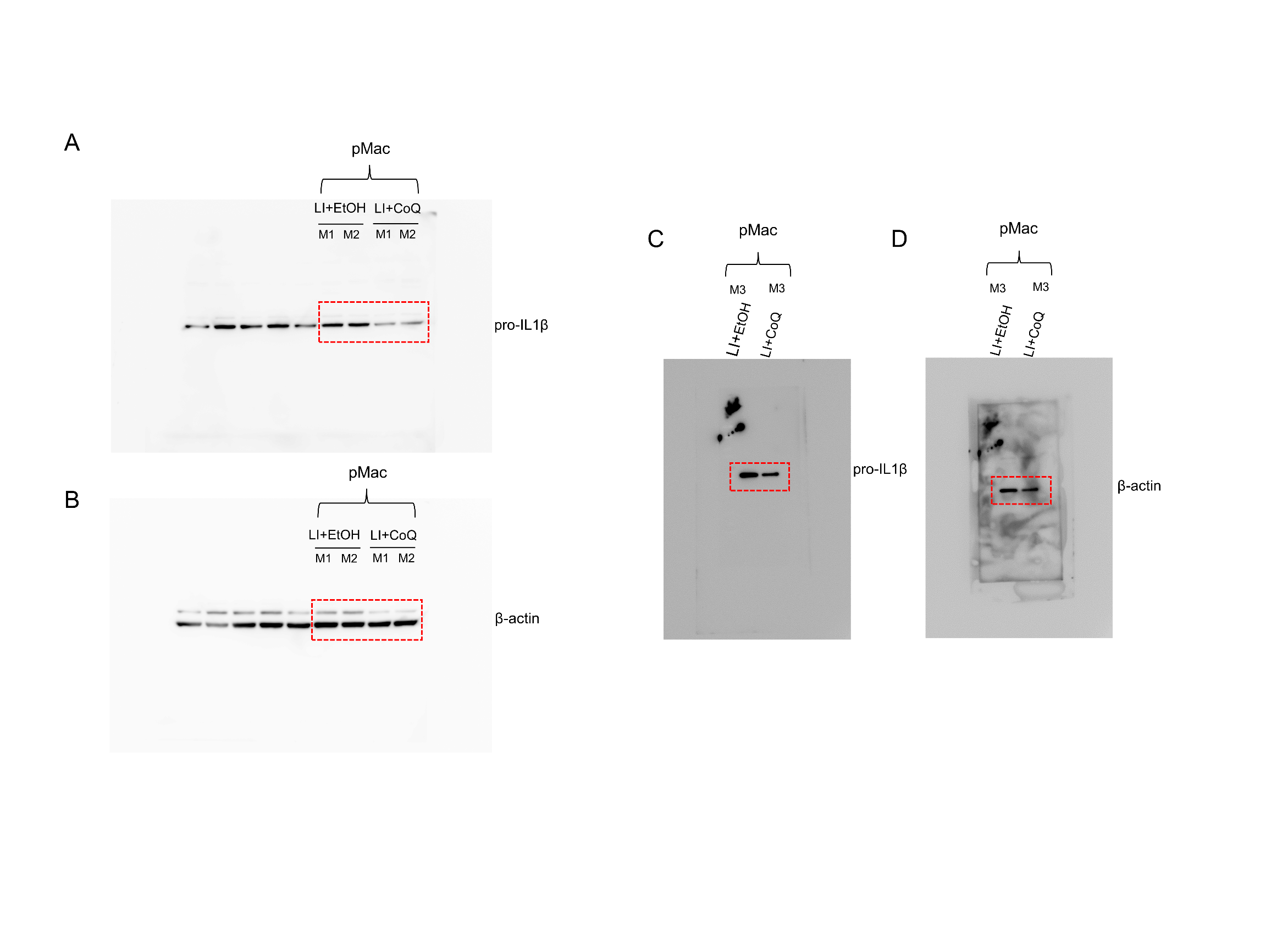


Supplemental Figure 11. Original images of Western blot (pro-IL1β and β-actin). (A)(B) On the same membrane shown pro-IL1β and β-actin. (C)(D) On the same membrane shown pro-IL1β and β-actin (The membrane cut prior to hybridization with antibodies). pMac, peritoneal macrophages; M, mice; LI, lipopolysaccharide+interferon γ; EtOH, ethanol; CoQ, Coenzyme Q10.


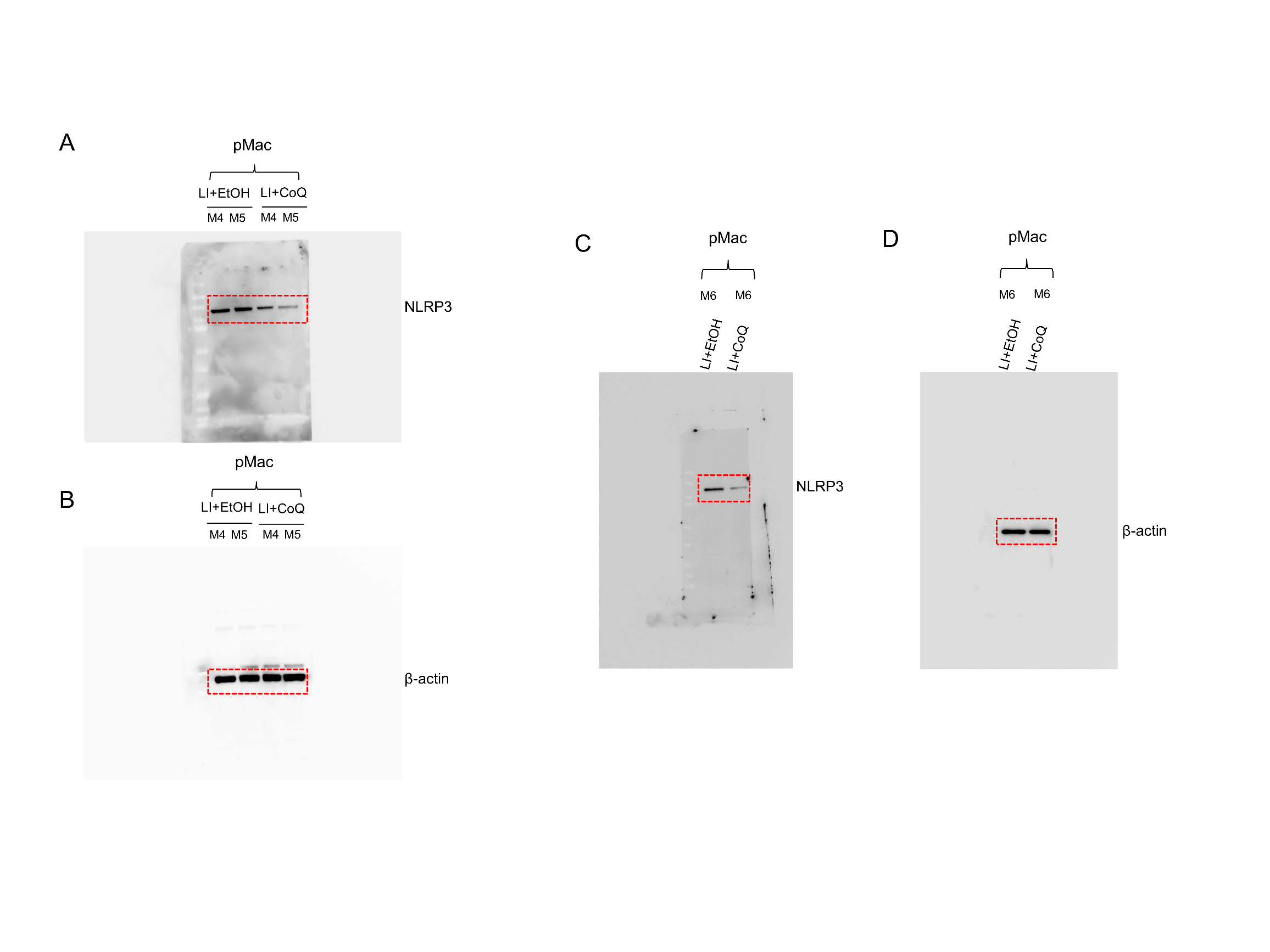


Supplemental Figure 12. Original images of Western blot (NLRP3 and β-actin). (A)(B) On the same membrane shown NLRP3 and β-actin (The membrane cut prior to hybridisation with antibodies). (C)(D) On the same membrane shown NLRP3 and β-actin (The membrane cut prior to hybridisation with antibodies). pMac, peritoneal macrophages; M, mice; LI, lipopolysaccharide+interferon γ; EtOH, ethanol; CoQ, Coenzyme Q10.


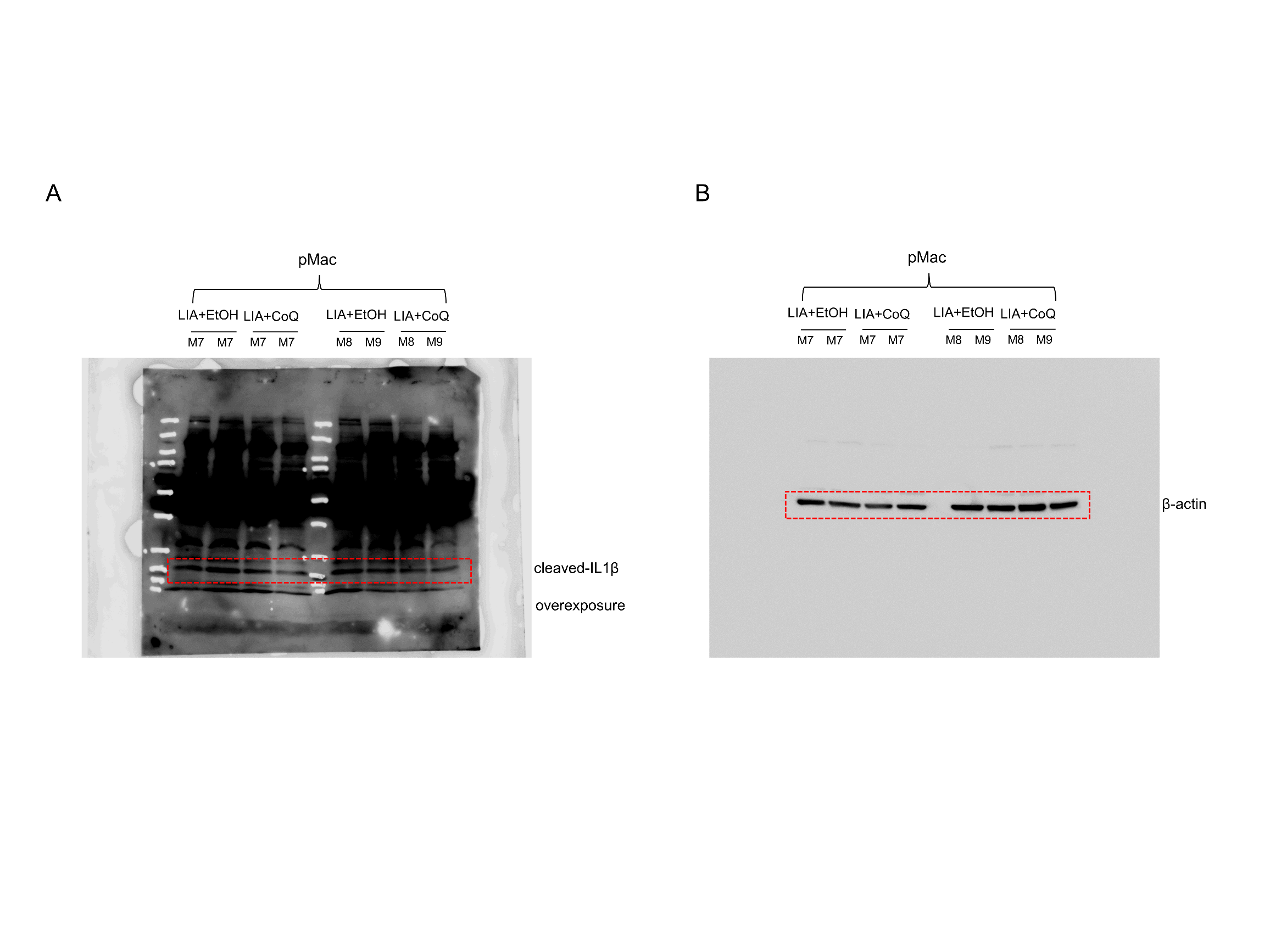


Supplemental Figure 13. Original images of Western blot (cleaved-IL1β and β-actin). (A)(B) On the same membrane shown cleaved-IL1β and β-actin. pMac, peritoneal macrophages; M, mice; LIA, lipopolysaccharide+interferon γ+adenosine triphosphate; EtOH, ethanol; CoQ, Coenzyme Q10.

**Supplemental Tables**

Supplemental Table 1. Baseline characteristics of MI patients in Control and CoQ10 groups.

| **Variable** | **Control group（n=59）** | **CoQ10 group（n=61）** | ***p-value*** |
| --- | --- | --- | --- |
| Age（year） | 59.05±13.47 | 57.11±11.30 | 0.395 |
| Height (m) | 1.63±0.07 | 1.65 ±0.08 | 0.350 |
| Weight (kg) | 67.44±11.38 | 69.77±13.83 | 0.317 |
| BMI (kg/m^2^) | 25.16(22.86-26.44) | 25.28(23.37-26.82) | 0.652 |
| Sex (M/F) | 50/9 | 52/9 | 0.939 |
| Current smoking[*n*(%)] | 26 (44.06) | 27 (44.26) | 0.983 |
| Current drinking[*n*(%)] | 31 (52.54) | 28 (45.90) | 0.467 |
| Hypertension[*n*(%)] | 33 (55.93) | 29 (47.54) | 0.358 |
| SBP (mmHg) | 117.00(106.00-132.00) | 122.00(107.50-135.00) | 0.271 |
| DBP (mmHg) | 71.00(69.00-79.00) | 77.00(67.25-87.50) | 0.139 |
| HR (bpm) | 77.00(71.00-87.00) | 79.00 (72.00-90.00) | 0.696 |
| Diabetes[*n*(%)] | 7 (11.86) | 11 (18.03) | 0.344 |
| HbA1c (%) | 6.20 (5.80-7.10) | 6.30 (5.92-6.80) | 0.498 |
| Hyperlipidemia [*n*(%)] | 15 (25.42) | 20 (32.78) | 0.375 |
| TC (mmol/L) | 4.74±1.17 | 4.57±1.05 | 0.421 |
| TG (mmol/L) | 1.35 (0.97-2.19) | 1.55 (1.09-2.27) | 0.498 |
| LDL-C (mmol/L) | 2.50±0.85 | 2.48±0.73 | 0.878 |
| HDL-C (mmol/L) | 1.01 (0.88-1.17) | 1.05 (0.89-1.22) | 0.703 |
| Renal insufficiency [*n*(%)] | 5 (8.47) | 7 (11.47) | 0.584 |
| Scr (μmol/L) | 70.80(61.00-87.50) | 75.40(66.25-83.30) | 0.295 |
| BUN (mmol/L) | 5.56 (4.80-6.99) | 5.64 (4.29-6.71) | 0.451 |
| Cys-C (mg/L) | 1.09 (0.94-1.20) | 1.03(0.83-1.20) | 0.476 |
| eGFR (%) | 90.00(73.00-105.00) | 93.00(79.75-100.00) | 0.864 |
| UA (mmol/L) | 363.47±103.54 | 360.73±104.73 | 0.885 |
| STEMI [*n*(%)] | 50 (84.74) | 48 (78.68) | 0.391 |
| NSTEMI [*n*(%)] | 9 (15.25) | 13 (21.31) | 0.391 |
| Killip classification | 1.00 (1.00-1.00) | 1.00 (1.00-2.00) | 0.062 |
| GRACE | 146.50±25.27 | 140.36±35.51 | 0.276 |
| Number of stenosis vessel | 2.00 (1.00-3.00) | 2.00 (1.00-2.75) | 0.237 |
| Culprit vessel |  |  |  |
| LAD | 35 (59.32) | 29 (47.54) | 0.196 |
| LCX | 12(20.33) | 14 (22.95) | 0.728 |
| RC | 18 (30.50) | 21 (34.42) | 0.647 |
| LV (mm) | 45.59±3.69 | 45.83±5.85 | 0.787 |
| LVEF (%) | 60.00(58.00-64.00) | 60.00(54.00-63.75) | 0.113 |
| LVFS (%) | 32.00(30.00-34.00) | 30.00(28.00-34.00) | 0.142 |
| Aspirin + Ticagrelor [*n*(%)] | 35 (59.32) | 40 (65.57) | 0.479 |
| Aspirin + Clopidogrel [*n*(%)] | 24 (40.67) | 21 (34.42) | 0.479 |
| Statins [*n*(%)] | 59 (100) | 61 (100) | - |
| β-blockers | 50 (84.74) | 56 (91.80) | 0.229 |
| ACEI/ARB [*n*(%)] | 37 (62.71) | 42 (68.85) | 0.478 |
| CoQ10 [*n*(%)] | 0 (0) | 61 (100) | - |

BMI, body mass index; SBP, systolic blood pressure; DBP, diastolic blood pressure; HR, heart rate; TC, total cholesterol; TG, triglyceride; LDL-C, low density lipoprotein cholesterol; HDL-C, high density lipoprotein cholesterol; Scr, Serum creatinine; BUN, blood urea nitrogen; Cys-C, Cystatin C; eGFR, estimated glomerular filtration rate; UA, uric acid; STEMI, ST-Elevation Myocardial Infarction; NSTEMI, non-ST-Elevation Myocardial Infarction; GRACE, global registered risk score for acute coronary events; LAD: left anterior descending artery; LCX: left circumflex artery; RC: right coronary artery; LV, left ventricular end diastolic dimension; LVEF, left ventricular ejection fraction; LVFS, left ventricular fractional shortening; ACEI/ARB: angiotensin converting enzyme inhibitor/angiotensin receptor blocker; CoQ10, coenzyme Q10.

Supplemental Table 2. Antibodies used for flow cytometry analyses.

| **Antibodies** | **Fluorophore** | **Dilution ratio** | **Clone** | **Source** | **Identifier** |
| --- | --- | --- | --- | --- | --- |
| Anti-CD45.2 | Percp-Cy5.5 | 1:125 | 104 | Biolegend | Cat# 109827 |
| Anti-Ly6G | PE-Cy7 | 1:300 | 1A8 | BioLegend | Cat# 127617 |
| Anti-CD64 | APC | 1:300 | X54-5/7.1 | Biolegend | Cat# 139305 |
| Anti-Ly6C | FITC | 1:300 | HK1.4 | BioLegend | Cat# 128005 |
| Anti-CCR2 | BV421 | 1:250 | SA203G11 | Biolegend | Cat# 150605 |
| Anti-IL1β | PE | 1:300 | 12-7114-82 | eBioscience | NJTEN3 |

Supplemental Table 3. Primers for mouse gene expression.

| **Gene Name** | **Species** | **Forward** | **Reverse** |
| --- | --- | --- | --- |
| *36b4* | *Mus Musculus* | 5’-GCTCCAAGCAGATGCAGCA-3’ | 5’-CCGGATGTGAGGCAGCAG-3’ |
| *Nlrp3* | *Mus Musculus* | 5’-ATTACCCGCCCGAGAAAGG-3’ | 5’-TCGCAGCAAAGATCCACACAG-3’ |
| *Caspase1* | *Mus Musculus* | 5’-ACAAGGCACGGGACCTATG-3’ | 5’-TCCCAGTCAGTCCTGGAAATG-3’ |
| *Il1β* | *Mus Musculus* | 5’-TGACAGTGATGAGAATGACCTGTT-3’ | 5’-TTGGAAGCAGCCCTTCATCT-3’ |
| *Il18* | *Mus Musculus* | 5’-CAAACCTTCCAAATCACTTCCT-3’ | 5’-TCCTTGAAGTTGACGCAAGA-3’ |
| *Tnfα* | *Mus Musculus* | 5’-CGGAGTCCGGGCAGG-3’ | 5’-GCTGGGTAGAGAATGGATGAA-3’ |
| *Il6* | *Mus Musculus* | 5’-GCTACCAAACTGGATATAATCAGGA-3’ | 5’-CCAGGTAGCTATGGTACTCCAGAA-3’ |
| *Ccl2* | *Mus Musculus* | 5’-CAGCCAGATGCAGTTAACGC-3’ | 5’-GCCTACTCATTGGGATCATCTTG-3’ |
| *iNOS* | *Mus Musculus* | 5’-AGACCTCAACAGAGCCCTCA-3’ | 5’-GCAGCCTCTTGTCTTTGACC-3’ |

Supplemental Table 4. Concentration of plasma CoQ10 data between healthy controls and MI patients. (As shown in Figure 1B)

|  |  | Healthy controls (n=11) |  | MI patients (n=11) | |
| --- | --- | --- | --- | --- | --- |
| Plasma CoQ10(μg/ml) |  | 0.76±0.31 |  | 0.46±0.10 |  |

CoQ10, Coenzyme Q10.

Supplemental Table 5. Change value of echocardiographic and LnBNP of MI patients at 1-month and 3-month post-PCI. (As shown in Figure 1C, Figure 1D)

|  | | MI patients | |
| --- | --- | --- | --- |
|  |  | Blank control group (n=59) | CoQ10 group (n=61) |
| 1-month | △EF (%) | 0.00[(-5.00)-(5.00)] | 3.00[(-1.50)-(8.00)] |
|  | △FS (%) | 0.00[(-2.00)-(4.00)] | 2.00(0.00-6.00) |
|  | △LnBNP | -0.37[(-1.21)-(0.39)] | -0.65[(-1.70)-(0.00)] |
|  | △EF (%) | -0.10[(-7.00)-(3.00)] | 2.00[(-2.50)-(9.00)] |
| 3-month | △FS (%) | 0.00[(-4.00)-(3.00)] | 2.00[(-1.50)-(6.00)] |
|  | △LnBNP | -0.81[(-1.75)-(0.87)] | -1.52[(-2.78)-(-0.40)] |

EF, ejection fraction; FS, fractional shortening; BNP, brain natriuretic peptide; CoQ10, Coenzyme Q10.

**The 1^st^ batch of animal experiments (Pilot study)**

Supplemental Table 6. The data of the pilot study for evaluating various dissolution and administration methods of CoQ10 in mice. (As shown in Supplemental Figure 2)

|  | Control (n=3) | Corn oil+CoQ10 (i.g., n=3) | Corn oil+CoQ10 (i.p., n=3) | Tween80+CoQ10 (i.g., n=3) |
| --- | --- | --- | --- | --- |
| Plasma concentration of CoQ10（ng/ml） | 1.05±0.30 | 6.81±2.71 | 4.10±0.46 | 4.32±3.88 |
| Notes: In each group, 3 mice were used, and no mice died during the experiment. i.g.: gastric gavage; i.p.: intraperitoneal injection. CoQ10, Coenzyme Q10. | | | | |

**The 2^nd^ batch of animal experiments (survival analysis)**

Supplemental Table 7. Survival data of mice administrated with Vehicle or CoQ10 following LAD ligation after 28 days. ( As shown in Figure 2A)

|  |  | MI+Vehicle (n=21) | |  | MI+CoQ10 (n=21) | |
| --- | --- | --- | --- | --- | --- | --- |
| Number (n) |  | dead | alive |  | death | alive |
|  |  | 12 | 9 |  | 8 | 13 |
| Notes:  (1) In the MI+Vehicle group, 25 mice were subjected to LAD ligation, and 4 died during the first 24 hours;  (2) In the MI+CoQ10 group, 1 died of suffocation by gavage, 24 mice were subjected to LAD ligation, and 3 died during the first 24 hours. | | | | | | |

**The 3^rd^ batch of animal experiments (echocardiography, WGA staining, Masson’s trichrome staining, Serum BNP levels, immunofluorescence staining)**

Supplemental Table 8. Echocardiographic data of mice at 28 days after sham or LAD ligation surgeries. (As shown in Figure 2C)

|  | Sham | |  | MI | |
| --- | --- | --- | --- | --- | --- |
|  | Vehicle (n=5) | CoQ10 (n=5) |  | Vehicle (n=6) | CoQ10 (n=7) |
| EF (%) | 77.21±2.12 | 77.92±2.15 |  | 19.94±5.66 | 30.12±7.23 |
| LVSV (μl) | 7.27±1.36 | 7.28±1.39 |  | 94.23±42.60 | 52.47±26.84 |
| LVDV (μl) | 33.35±5.39 | 35.10±3.56 |  | 135.49±40.97 | 93.68±28.14 |
| Notes:  (1) In the Sham+Vehicle group, 6 mice underwent a sham operation, and 1 died during the experiment. Finally, 5 mice entered the stage of result analysis;  (2) In the Sham+CoQ10 group, 6 mice underwent a sham operation, and 1 died during the experiment. Finally, 5 mice entered the stage of result analysis;  (3) In the MI+vehicle group, 14 mice underwent LAD ligation operation, 2 died during the first 24 hours, and 6 died during the experiment. Finally, 6 mice entered the stage of result analysis;  (4) In the MI+CoQ10 group, 13 mice underwent LAD ligation operation, 2 died during the first 24 hours, and 4 died during the experiment. Finally, 7 mice entered the stage of result analysis. | | | | | |

Supplemental Table 9. WGA staining data of mice at 28 days after sham or LAD ligation surgeries. (As shown in Figure 2E)

|  | Sham | |  | MI | |
| --- | --- | --- | --- | --- | --- |
|  | Vehicle (n=5) | CoQ10 (n=5) |  | Vehicle (n=6) | CoQ10 (n=7) |
| CSA | 1.00±0.03 | 0.98±0.07 |  | 1.32±0.11 | 1.05±0.08 |
| Notes:  (1) In the Sham+Vehicle group, 6 mice underwent a sham operation, and 1 died during the experiment. Finally, 5 mice entered the stage of result analysis;  (2) In the Sham+CoQ10 group, 6 mice underwent a sham operation, and 1 died during the experiment. Finally, 5 mice entered the stage of result analysis;  (3) In the MI+vehicle group, 14 mice underwent LAD ligation operation, 2 died during the first 24 hours, and 6 died during the experiment. Finally, 6 mice entered the stage of result analysis;  (4) In the MI+CoQ10 group, 13 mice underwent LAD ligation operation, 2 died during the first 24 hours, and 4 died during the experiment. Finally, 7 mice entered the stage of result analysis. | | | | | |

Supplemental Table 10. Masson’s trichrome staining data of mice at 28 days after sham or LAD ligation surgeries. (As shown in Figure 2G)

|  | Sham | |  | MI | |
| --- | --- | --- | --- | --- | --- |
|  | Vehicle (n=5) | CoQ10 (n=5) |  | Vehicle (n=6) | CoQ10 (n=6) |
| Fibrotic area (%) | 3.22±2.16 | 2.17±1.29 |  | 17.60±1.76 | 11.95±6.58 |
| Notes:  (1) In the Sham+Vehicle group, 6 mice underwent a sham operation, and 1 died during the experiment. Finally, 5 mice entered the stage of result analysis;  (2) In the Sham+CoQ10 group, 6 mice underwent a sham operation, and 1 died during the experiment. Finally, 5 mice entered the stage of result analysis;  (3) In the MI+vehicle group, 14 mice underwent LAD ligation operation, 2 died during the first 24 hours, and 6 died during the experiment. Finally, 6 mice entered the stage of result analysis;  (4) In the MI+CoQ10 group, 13 mice underwent LAD ligation operation, 2 died during the first 24 hours, and 4 died during the experiment, 1 exhibited fragmentation and damage of the infarcted area in cardiac tissue slices. Finally, 6 mice entered the stage of result analysis. | | | | | |

Supplemental Table 11. Serum BNP levels data of mice at 28 days after sham or LAD ligation surgeries. (As shown in Figure 2H)

|  | Sham | |  | MI | |
| --- | --- | --- | --- | --- | --- |
|  | Vehicle (n=4) | CoQ10 (n=4) |  | Vehicle (n=5) | CoQ10 (n=5) |
| Serum BNP levels (pg/ml) | 176.23±20.60 | 152.04±7.75 |  | 205.92±33.63 | 167.68±16.97 |
| Notes:  (1) In the Sham+Vehicle group, 6 mice underwent a sham operation, 1 died during the experiment, and 1 failed to collect enough blood. Finally, 4 mice entered the stage of result analysis;  (2) In the Sham+CoQ10 group, 6 mice underwent a sham operation, 1 died during the experiment, and 1 appeared hemolysis. Finally, 4 mice entered the stage of result analysis;  (3) In the MI+vehicle group, 14 mice underwent LAD ligation operation, 2 died during the first 24 hours, 6 died during the experiment, and 1 appeared hemolysis. Finally, 5 mice entered the stage of result analysis;  (4) In the MI+CoQ10 group, 13 mice underwent LAD ligation operation, 2 died during the first 24 hours, 4 died during the experiment, 1 failed to collect enough blood, and 1 appeared hemolysis. Finally, 5 mice entered the stage of result analysis. | | | | | |

Supplemental Table 12. Immunofluorescence staining (IL1β and Mac3) data of mice at 28 days after LAD ligation surgeries. (As shown in Figure 4B)

|  | MI | |
| --- | --- | --- |
|  | Vehicle (n=6) | CoQ10 (n=7) |
| IL1β positive Mac (%) | 72.00±6.35 | 57.24±11.61 |
| Notes:  (1) In the MI+vehicle group, 14 mice underwent LAD ligation operation, 2 died during the first 24 hours, and 6 died during the experiment. Finally, 6 mice entered the stage of result analysis;  (2) In the MI+CoQ10 group, 13 mice underwent LAD ligation operation, 2 died during the first 24 hours, and 4 died during the experiment. Finally, 7 mice entered the stage of result analysis. | | |

Supplemental Table 13. Immunofluorescence staining (ROS and Mac3) data of mice at 28 days after LAD ligation surgeries. (As shown in Supplemental Figure 4B)

|  | MI | |
| --- | --- | --- |
|  | Vehicle (n=6) | CoQ10 (n=7) |
| ROS positive Mac (%) | 72.62±3.79 | 60.48±9.36 |
| Notes:  (1) In the MI+vehicle group, 14 mice underwent LAD ligation operation, 2 died during the first 24 hours, and 6 died during the experiment. Finally, 6 mice entered the stage of result analysis;  (2) In the MI+CoQ10 group, 13 mice underwent LAD ligation operation, 2 died during the first 24 hours, and 4 died during the experiment. Finally, 7 mice entered the stage of result analysis. | | |

**The 4^th^ batch of animal experiments**

Supplemental Table 14. Flow cytometry data of the left ventricles at 28 days after sham/LAD ligation surgeries. (As shown in Figure 2I)

|  | Sham | |  | MI | |
| --- | --- | --- | --- | --- | --- |
|  | Vehicle (n=8) | CoQ10 (n=8) |  | Vehicle (n=10) | CoQ10 (n=11) |
| Mac (×10^4^) | 5.28±0.83 | 2.63±0.67 |  | 14.10±4.07 | 7.80±4.37 |
| Neut (×10^3^) | 3.72±1.92 | 3.85±2.38 |  | 2.32±1.08 | 1.66±0.74 |
| Mono (×10^3^) | 1.37±0.36 | 0.84±0.34 |  | 9.63±5.20 | 3.51±1.51 |
| CCR2^+^ Mac(×10^4^) | 1.13±0.55 | 0.25±0.11 |  | 2.65±0.87 | 1.63±0.48 |
| Notes:  (1) In the Sham+Vehicle group, 9 mice underwent a sham operation, and 1 died during the experiment. Finally, 8 mice entered the stage of result analysis;  (2) In the Sham+CoQ10 group, 9 mice underwent a sham operation, and 1 died during the experiment. Finally, 8 mice entered the stage of result analysis;  (3) In the MI+vehicle group, 18 mice underwent LAD ligation operation, 1 died during the first 24 hours, and 7 died during the experiment. Finally, 10 mice entered the stage of result analysis;  (4) In the MI+CoQ10 group, 16 mice underwent LAD ligation operation; 1 died during the first 24 hours, and 4 died during the experiment. Finally, 11 mice entered the stage of result analysis. | | | | | |

**The 5^th^ batch of animal experiments**

Supplemental Table 15. Flow cytometry data of the left ventricles at 3 days after sham/LAD ligation surgeries. (As shown in Supplemental Figure 3)

|  | Sham | |  | MI | |
| --- | --- | --- | --- | --- | --- |
|  | Vehicle (n=7) | CoQ10 (n=3) |  | Vehicle (n=3) | CoQ10 (n=3) |
| Mac (×10^5^) | 0.89±0.12 | 1.53±0.65 |  | 4.63±0.31 | 3.40±0.20 |
| Neut (×10^5^) | 0.24±0.11 | 0.46±0.15 |  | 3.09±1.17 | 2.06±1.41 |
| Mono (×10^5^) | 0.10±0.05 | 0.09±0.01 |  | 2.05±1.02 | 1.30±0.23 |
| CCR2^+^ Mac (×10^5^) | 0.53±0.09 | 0.97±0.36 |  | 2.72±0.26 | 1.96±0.30 |
| Notes:  (1) In the Sham+Vehicle group, 7 mice underwent a sham operation, and no mice died during the experiment. Finally, 7 mice entered the stage of result analysis;  (2) In the Sham+CoQ10 group, 3 mice underwent a sham operation, and no mice died during the experiment. Finally, 3 mice entered the stage of result analysis;  (3) In the MI+vehicle group, 6 mice underwent LAD ligation operation, 1 died during the first 24 hours, and 2 died during the experiment. Finally, 3 mice entered the stage of result analysis;  (4) In the MI+CoQ10 group, 6 mice underwent LAD ligation operation, 2 died during the first 24 hours, and 1 died during the experiment. Finally, 3 mice entered the stage of result analysis. | | | | | |

**The 6^th^ batch of animal experiments**

Supplemental Table 16. Mean fluorescence intensity (MFI) data of IL-1β in the CCR2^+^ macrophages of mice 3 days after LAD ligation. (As shown in Figure 4E)

|  | MI | |
| --- | --- | --- |
|  | Vehicle (n=6) | CoQ10 (n=7) |
| MFI | 333.33±53.58 | 244.71±80.79 |
| Notes:  (1) In the MI+vehicle group, 8 mice underwent LAD ligation operation, 1 died during the first 24 hours, and 1 died during the experiment. Finally, 6 mice entered the stage of result analysis;  (2) In the MI+CoQ10 group, 8 mice underwent LAD ligation operation, and 1 died during the experiment. Finally, 7 mice entered the stage of result analysis. | | |

**The 7^th^ batch of animal experiments**

Supplemental Table 17. Gene expression data of the infarct myocardium at 3 days after sham/LAD ligation surgeries. (As shown in Figure 4A, Supplemental Figure 8)

|  | Sham | |  | MI | |
| --- | --- | --- | --- | --- | --- |
|  | Vehicle (n=3) | CoQ10 (n=3) |  | Vehicle (n=5) | CoQ10 (n=5) |
| *Il1β* | 1.04±0.34 | 0.37±0.11 |  | 9.63±2.05 | 6.04±1.25 |
| *Il6* | 1.05±0.99 | 0.17±0.12 |  | 31.37±14.93 | 31.24±11.37 |
| *Tnfα* | 1.05±0.39 | 0.54±0.12 |  | 5.40±0.81 | 3.23±0.65 |
| *Ccl2* | 1.03±0.36 | 0.67±0.23 |  | 25.12±3.57 | 16.95±5.47 |
| *Nlrp3* | 1.03±0.32 | 0.72±0.12 |  | 4.33±0.89 | 3.30±0.88 |
| *Caspase1* | 1.00±0.12 | 0.62±0.13 |  | 2.39±0.30 | 1.68±0.59 |
| *Il18* | 1.00±0.30 | 0.75±0.17 |  | 4.23±1.33 | 2.41±0.70 |
| *iNOS(NOS2)* | 1.05±0.38 | 1.17±0.14 |  | 2.31±0.42 | 1.82±0.34 |
| Notes:  (1) In the Sham+Vehicle group, 5 mice underwent a sham operation; 1 died during the first 24 hours, and 1 died during the experiment. Finally, 3 mice entered the stage of result analysis;  (2) In the Sham+CoQ10 group, 5 mice underwent a sham operation, and 2 died during the first 24 hours. Finally, 3 mice entered the stage of result analysis;  (3) In the MI+vehicle group, 8 mice underwent LAD ligation operation, 0 died during the first 24 hours, and 3 died during the experiment. Finally, 5 mice entered the stage of result analysis;  (4) In the MI+CoQ10 group, 8 mice underwent LAD ligation operation; 1 died during the first 24 hours, and 2 died during the experiment. Finally, 5 mice entered the stage of result analysis. | | | | | |

**The 8^th^ batch of animal experiments**

Supplemental Table 18. Gene expression data of the infarct myocardium at 28 days after sham/LAD ligation surgeries. (As shown in Supplemental Figure 9)

|  | Sham | |  | MI | |
| --- | --- | --- | --- | --- | --- |
|  | Vehicle (n=5) | CoQ10 (n=5) |  | Vehicle (n=6) | CoQ10 (n=7) |
| *Il1β* | 1.00±0.42 | 0.99±0.40 |  | 2.27±1.14 | 1.44±0.53 |
| *Il6* | 1.05±0.44 | 1.08±0.46 |  | 12.34±6.26 | 5.94±3.46 |
| *Tnfα* | 1.05±0.44 | 1.08±0.46 |  | 4.61±2.18 | 2.01±0.48 |
| *Ccl2* | 1.04±0.66 | 1.02±0.60 |  | 2.34±1.01 | 1.49±0.40 |
| *Nlrp3* | 1.03±0.62 | 1.23±0.47 |  | 5.32±2.91 | 2.19±0.73 |
| *Caspase1* | 1.00±0.11 | 1.15±0.23 |  | 2.39±0.55 | 1.47±0.23 |
| *Il18* | 1.03±0.51 | 1.07±0.57 |  | 1.94±0.70 | 1.23±0.68 |
| *iNOS(Nos2)* | 1.02±0.29 | 1.11±0.42 |  | 1.87±0.46 | 1.44±0.38 |
| Notes:  (1) In the Sham+Vehicle group, 6 mice underwent a sham operation, and 1 died during the experiment. Finally, 5 mice entered the stage of result analysis;  (2) In the Sham+CoQ10 group, 6 mice underwent a sham operation, and 1 died during the first 24 hours. Finally, 5 mice entered the stage of result analysis;  (3) In the MI+vehicle group, 12 mice underwent LAD ligation operation, 1 died during the first 24 hours, and 5 died during the experiment. Finally, 6 mice entered the stage of result analysis;  (4) In the MI+CoQ10 group, 12 mice underwent LAD ligation operation, 2 died during the first 24 hours, and 3 died during the experiment. Finally, 7 mice entered the stage of result analysis. | | | | | |

**The 9^th^ batch of animal experiments**

Supplemental Table 19. Gene expression data of CCR2^+^ macrophages (using flow cytometry sorting) in the infarct myocardium of mice at 3 days after LAD-ligation surgeries. (As shown in Supplemental Figure 10)

|  | MI | |
| --- | --- | --- |
|  | Vehicle (n=7) | CoQ10 (n=7) |
| *Il1β* | 0.98±0.32 | 0.45±0.16 |
| *Il6* | 1.32±0.95 | 0.32±0.13 |
| *Tnfα* | 1.18±0.66 | 0.41±0.25 |
| *Ccl2* | 1.07±0.43 | 0.73±0.14 |
| *Nlrp3* | 1.09±0.44 | 0.44±0.21 |
| *Caspase1* | 1.06±0.36 | 0.72±0.29 |
| *Il18* | 1.18±0.64 | 0.42±0.16 |
| *iNOS(Nos2)* | 2.67±2.98 | 0.41±0.40 |
| Notes:  (1) In the MI+vehicle group, 8 mice underwent LAD ligation operation; 0 died during the first 24 hours, and 1 died during the experiment. Finally, 7 mice entered the stage of result analysis;  (2) In the MI+CoQ10 group, 7 mice underwent LAD ligation operation, 0 died during the first 24 hours, and 0 died during the experiment. Finally, 7 mice entered the stage of result analysis. | | |

Supplemental Table 20. Gene expression data of peritoneal macrophages with or without stimulation of LPS/INFγ and in the presence or absence of CoQ10. (As shown in Figure 3A, Supplemental Figure 6)

|  | LPS (-) + FNγ (-) | |  | LPS (+) + IFNγ (+) | |
| --- | --- | --- | --- | --- | --- |
|  | Vehicle (n=3) | CoQ10 (n=3) |  | Vehicle (n=3) | CoQ10 (n=3) |
| *Il6* | 1.03±0.34 | 0.94±0.59 |  | 27.90±1.22 | 8.51±3.45 |
| *Il1β* | 1.02±0.29 | 0.84±0.16 |  | 2205.77±233.09 | 1010.61±357.23 |
| *Tnfα* | 1.01±0.17 | 0.94±0.16 |  | 85.36±6.66 | 49.74±8.72 |
| *Nlrp3* | 1.01±0.11 | 1.00±0.09 |  | 12.92±1.21 | 7.88±1.43 |
| *Ccl2* | 1.01±0.22 | 0.95±0.12 |  | 10.08±2.78 | 6.11±1.71 |
| *Caspase1* | 1.02±0.47 | 1.55±0.56 |  | 5.57±0.99 | 3.09±1.55 |
| *Il18* | 1.02±0.66 | 1.70±1.49 |  | 309.72±60.97 | 191.64±44.64 |
| *iNOS(Nos2)* | 1.02±0.92 | 0.51±0.40 |  | 1431.63±577.90 | 607.79±425.55 |
| LPS, lipopolysaccharide; IFNγ, interferon γ.  Supplemental Table 21. FPKM data of peritoneal macrophages with or without stimulation of LPS/INFγ and in the presence or absence of CoQ10. (As shown in Supplemental Figure 7)   \|  \| LPS (-) + FNγ (-) \| \|  \| LPS (+) + IFNγ (+) \| \| \| --- \| --- \| --- \| --- \| --- \| --- \| \|  \| Vehicle (n=3) \| CoQ10 (n=3) \|  \| Vehicle (n=3) \| CoQ10 (n=3) \| \| *Il6* \| 0.05±0.05 \| 0.07±0.09 \|  \| 1.60±0.39 \| 0.45±0.16 \| \| *Il1β* \| 0.06±0.04 \| 0.03±0.01 \|  \| 93.09±19.50 \| 40.10±8.19 \| \| *Tnf* \| 3.03±1.07 \| 3.31±0.22 \|  \| 518.45±97.43 \| 269.72±32.82 \| \| *Nlrp3* \| 4.76±0.71 \| 4.72±0.73 \|  \| 61.30±8.98 \| 37.44±7.70 \| \| *Ccl2* \| 12.10±2.38 \| 12.95±3.08 \|  \| 121.83±19.88 \| 75.58±16.27 \| \| *Caspase1* \| 34.57±2.38 \| 33.77±1.34 \|  \| 178.59±3.23 \| 152.97±6.43 \| \| *Il18* \| 1.88±0.38 \| 2.06±0.14 \|  \| 6.07±0.38 \| 4.78±0.34 \| \| *iNOS(Nos2)* \| 0.00±0.00 \| 0.00±0.01 \|  \| 202.48±3.81 \| 98.20±10.49 \| | | | | | |

LPS, lipopolysaccharide; IFNγ, interferon γ; FPKM, fragments per kilobase of exon per million fragments mapped.

Supplemental Table 22. Data of intracellular ROS production in peritoneal macrophages with or without stimulation of LPS and in the presence or absence of CoQ10. (As shown in Supplemental Figure 4D)

|  | LPS (-) | | |  | LPS (+) | | |
| --- | --- | --- | --- | --- | --- | --- | --- |
| 6h | Vehicle (n=8) |  | CoQ10 (n=8) |  | Vehicle (n=8) |  | CoQ10 (n=8) |
|  | 53.75±23.37 |  | 35.79±37.08 |  | 283.52±76.59 |  | 95.42±26.48 |
| 12h | Vehicle (n=8) |  | CoQ10 (n=8) |  | Vehicle (n=10) |  | CoQ10 (n=8) |
|  | 38.93±38.35 |  | 18.98±10.86 |  | 407.20±76.93 |  | 158.33±45.04 |
| 18h | Vehicle (n=8) |  | CoQ10 (n=8) |  | Vehicle (n=10) |  | CoQ10 (n=10) |
|  | 56.94±35.64 |  | 27.81±25.60 |  | 895.81±225.34 |  | 309.11±186.40 |

LPS, lipopolysaccharide.

Supplemental Table 23. Western blot data in peritoneal macrophages after stimulation with LPS(+)+INFγ(+) /+ATP(+) and in the presence of vehicle or CoQ10. (As shown in Figure 3D, Figure 3E, Figure 3F)

|  | LPS(+) + IFNγ(+) | |  | LPS(+) + IFNγ(+)+ATP(+) | |
| --- | --- | --- | --- | --- | --- |
|  | Vehicle (n=3) | CoQ10 (n=3) |  | Vehicle(n= 3) | CoQ10 (n=3) |
| NLRP3 | 1.00±0.06 | 0.24±0.14 |  | N.A. | N.A. |
| pro-IL1β | 1.00±0.14 | 0.41±0.15 |  | N.A. | N.A. |
| Cleaved- IL1β | N.A. | N.A. |  | 1.00±0.25 | 0.51±0.09 |
| LPS, lipopolysaccharide; IFNγ, interferon γ; ATP, adenosine triphosphate; N.A., not applicable. | | | | | |
